# The Anti-DNA Antibodies: Their Specificities for Unique DNA Structures and Their Unresolved Clinical Impact—A System Criticism and a Hypothesis

**DOI:** 10.3389/fimmu.2021.808008

**Published:** 2022-01-11

**Authors:** Ole Petter Rekvig

**Affiliations:** ^1^ Section of Autoimmunity, Fürst Medical Laboratory, Oslo, Norway; ^2^ Department of Medical Biology, Faculty of Health Sciences, UiT The Arctic University of Norway, Tromsø, Norway

**Keywords:** systemic lupus erythematosus, anti-DNA antibodies, DNA structures, classification criteria, pathogenicity

## Abstract

Systemic lupus erythematosus (SLE) is diagnosed and classified by criteria, or by experience, intuition and traditions, and not by scientifically well-defined etiology(ies) or pathogenicity(ies). One central criterion and diagnostic factor is founded on theoretical and analytical approaches based on our imperfect definition of the term “The anti-dsDNA antibody”. “The anti-dsDNA antibody” holds an archaic position in SLE as a unique classification criterium and pathogenic factor. In a wider sense, antibodies to unique transcriptionally active or silent DNA structures and chromatin components may have individual and profound nephritogenic impact although not considered yet – not in theoretical nor in descriptive or experimental contexts. This hypothesis is contemplated here. In this analysis, our state-of-the-art conception of these antibodies is probed and found too deficient with respect to their origin, structural DNA specificities and clinical/pathogenic impact. Discoveries of DNA structures and functions started with Miescher’s Nuclein (1871), *via* Chargaff, Franklin, Watson and Crick, and continues today. The discoveries have left us with a DNA helix that presents distinct structures expressing unique operations of DNA. All structures are proven immunogenic! Unique autoimmune antibodies are described against e.g. ssDNA, elongated B DNA, bent B DNA, Z DNA, cruciform DNA, or individual components of chromatin. In light of the massive scientific interest in anti-DNA antibodies over decades, it is an unexpected observation that the spectrum of DNA structures has been known for decades without being implemented in clinical immunology. This leads consequently to a critical analysis of historical and contemporary evidence-based data and of ignored and one-dimensional contexts and hypotheses: i.e. “one antibody - one disease”. In this study radical viewpoints on the impact of DNA and chromatin immunity/autoimmunity are considered and discussed in context of the pathogenesis of lupus nephritis.

## Introduction

This theoretical study critically analyses immunology of DNA and chromatin. The discussion is basically immunological and unlinked from SLE, but elements of the syndrome is discussed, as chromatin autoimmunity is relevant to understand SLE in both historical and contemporary contexts^1^.

“For the current state of knowledge remains vague when history is not considered, just as history remains vague without substantive knowledge of the current state” (Ludwik Fleck^2^) (1).

The citation above is highly relevant as backdrop for this study. The central idea is to reconsider historical data on DNA and anti-dsDNA antibodies in light of contemporary prioritized insight.

### History and Scientific Impact of Antibodies to DNA

The first reports on antibodies against dsDNA appeared in 1938 and 1939 in context of bacterial infections (2–4). Two decades later they were described in SLE (5–8). Already here we observe a conflict between the current view that the antibodies are unique biomarkers for SLE [see a relevant contextual discussion of “biomarker” (9)] and the historical facts that the antibodies were first described in patients infected with bacteria. Today, the strong links between anti-DNA antibodies and infections and malignancies is not considered important in contemporary rheumatological contexts (**Table 1** presents the major critical elements in this study).

**Table 1:** Historical and contemporary definitions of DNA and anti-DNA antibodies.

An important reflection by Ludvik Fleck is a correction to our deficient considerations related to impact of DNA/chromatin and corresponding autoantibodies in clinical medicine: *“For the current state of knowledge remains vague when history is not considered, just as history remains vague without substantive knowledge of the current state”* (1). The analyses presented here reveal that current knowledge remains vague on central aspects. It is easy to document that central data from historical science are not considered in contemporary knowledge and documentation as outlined below: •Historical data unmistakably demonstrate that anti-dsDNA antibodies were first described in bacterial infections (1938, 1939) – not in SLE (1957). This is not considered in classification or diagnostic criteria, all of which uniformly inform that “The anti-dsDNA antibody” is specific for SLE: “one antibody – one disease”. •Historical data unmistakably demonstrate that multiple functional DNA structures have individual immunogenic potentials and consequently induce production of unique cognate anti-DNA antibodies. These are not considered in classification criteria, nor in discussions of pathogenicity of unique anti-DNA antibodies. Considered is just the misleading term “The anti-dsDNA antibody”: Again leading to the paradigm: “One antibody – one disease”. •Historical and recent data unmistakably demonstrate that anti-DNA and anti-chromatin antibodies execute their pathogenic potential by binding chromatin exposed *in situ* on one hand – other data argue that anti-DNA antibodies bind cross-reactive, intrinsic matrix or basement membrane constituents. •Till now, no collaborative and/or comparative studies have been performed across the different models of lupus nephritis. This should be regarded as a *sine qua none* to develop consistent causal therapies, meaning therapy aiming at preventing true scientifically verified pathogenic processes. We have today to accept that the processes are in conflict with each other with poor perspectives to be solved. •Also, there is today a strong need to understand the impact of the steadily increasing number of previous and contemporary classification criteria for SLE. They are not linked to each other in an etiological or pathogenetic context, and they define a large number of heterogenous clinical SLE phenotypes. This makes cohort studies on homogenous SLE phenotypes difficult. Likewise, anti-dsDNA antibodies represent a group of antibody specificities. We need to define what we test for, why, and by which assay principles in order to leave the silently accepted term “one antibody – one disease” behind. •In conclusion: there is a need to create a bases for new definitions of parameters that may define bases for future studies. Those studies must aim to increase our insight into what SLE classification criteria are, if they are linked through common processes, what the etiology encompasses, and what pathogenic pathways are fundamental in SLE.

Anti-DNA antibodies are, nevertheless, important and play informative and controversial roles in history of immunology (10–16), in studies of antibody diversity and immunoglobulin variable region structures and genetics (11, 17–21), in molecular biology (22–26) as well as in rheumatology, infections and in malignancies [see e.g. (12, 13, 25, 27–32)]. In contrast to the considerable amount of studies on phenomenological and basic aspects of anti-dsDNA antibodies^3^, we still do not definitively know the critical incitements that promote their production *in vivo*.

**Figure 1:**
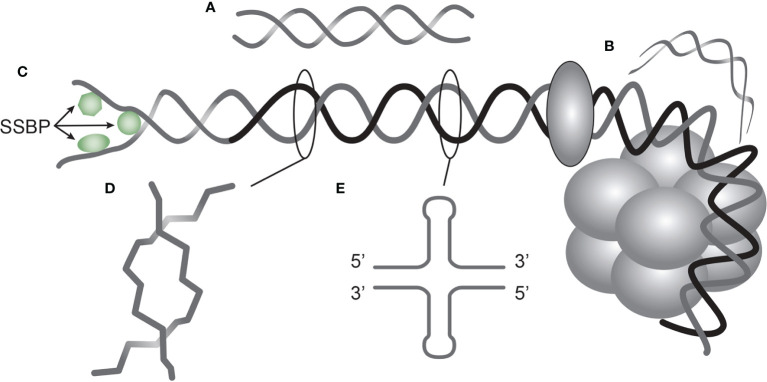
*DNA structures in chromatin express distinct DNA functions, and each structure is a unique antigen.* Elongated (linker) DNA is a relaxed, right-handed. low-energy linear form of B DNA **(A)**, while compacted B DNA as in plasmids (not shown) and in core nucleosomes are defined as bent B DNA **(B)**. In **(C)** the B DNA helix is opened by single-DNA binding proteins (i.e. proteins stabilizing ssDNA and polymerizes involved in replication and repair). In **(D)** Z DNA is demonstrated, which is a left-handed, high energy, supercoiled double helix. Physiologically, Z DNA forms during transcription as a result of torsional strain that depend on interaction of mobile polymerases. Z DNA is predominantly associated with linker DNA and regulate transcription. Cruciform DNA is another structure formed in dsDNA **(E)**, and is different from B and Z DNA. Its generation requires that repeat sequences (palindromes) in one strand is repeated on the other strand in opposite direction. The cruciforms are, like Z DNA, higher energy DNA structures. From an immunogenic point of view, each structure **(A–E)** is unique in terms of inducing highly specific antibodies with potential pathogenic impact if chromatin is exposed in situ. See text for details.

Furthermore, there is today not consensus on their targets *in vivo* – whether DNA (13) or non-DNA structures (33, 34). Important scientific data describing antibody specificity against functional DNA structures (**Figure 1**) are in current clinical immunology contexts largely neglected - but erroneously discussed in terms of avidity and not specificity (see below). This is not so in basic DNA research where structure and operation of individual forms of DNA are central elements to understand nature of DNA in biology (discussed below).

These short and decisive statements derive from a large series of studies, from preliminary conclusions, and from a categorical lack of international consensus. This is documented in hypotheses and theory studies [see e.g. (12, 35, 36)]. Conversely, the “Anti-dsDNA antibody” has achieved an aristocratic and time wise pompous status as a diagnostic and a pathogenic factor: See e.g. the Wikipedia-statement: “*Anti-dsDNA antibodies are incredibly specific for SLE* ^4^ *”.* This statement is from historical and contemporary concepts and data difficult to understand, because the data on the antibodies in non-SLE conditions are neglected or overlooked.

**Figure 2:**
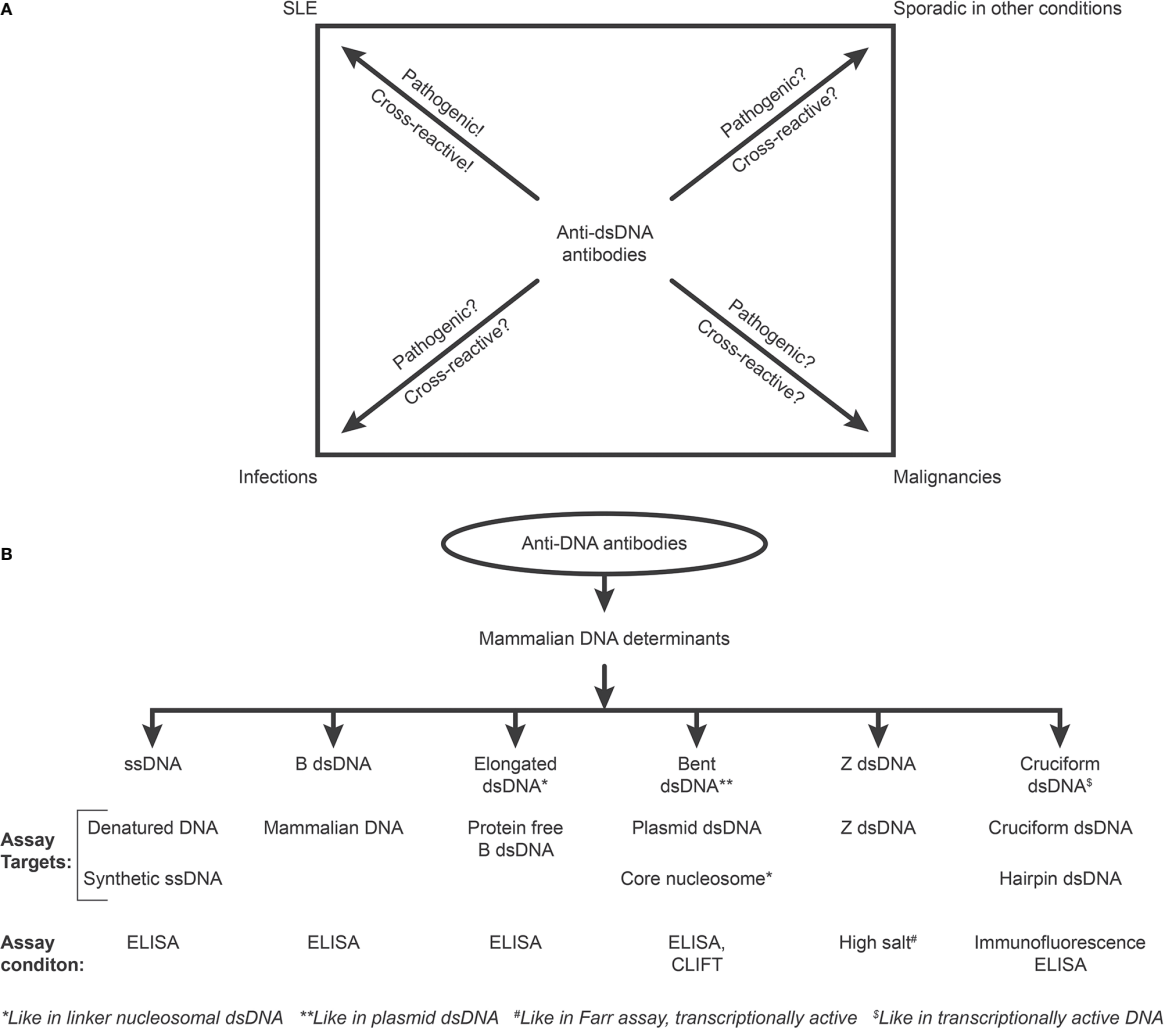
Critical questions related to the SLE classification criterion “The anti-dsDNA antibody” (criterion 11 in ACR) or “Anti-dsDNA” (criterion 6, Immunological criteria, SLICC). Principal problems are linked to the inadequate terminology of the anti-dsDNA antibodies. One problem [demonstrated in **(A)**] illustrates that “The anti-dsDNA antibody” is not unique for SLE, but appears regularly in context of infections, malignancies, and sporadic in other conditions. Little is examined whether anti-dsDNA antibodies are pathogenic and cross-reactive in the latter conditions [question marks in **(A)**], as they truly are in SLE. The second dominant problem considered for the “Anti-dsDNA antibody” is that the antibodies are presented as if “it” is monospecific for dsDNA without further specifications of target DNA structures. This has over decades crystallized the conception that different assay systems detect antibodies possessing different avidities but not different specificities! This conflict is principally [demonstrated in **(B)**]. The “ssDNA/dsDNA” structures are categorized in 6 main sets. Antibodies against all these dsDNA structures have been identified by conventional assay systems, like ELISA in physiological salt (ssDNA, B DNA, elongated B DNA, bent B dsDNA), in high salt (Z dsDNA), and cruciform dsDNA in addition to heterogeneous binding to proteins and phospholipids. The idiom that anti-dsDNA antibodies bind dsDNA in a singular form as in the ACR or SLICC classification systems must be challenged by the multifaceted recognition pattern of anti-dsDNA antibodies as informed about in **(B)** Thus, data in this figure require that assay systems for anti-dsDNA antibodies relates to categorized structural DNA specificities. Lack of implementation of the structural and molecular recognition pattern recognized by individual anti-dsDNA antibodies undermine the potential clinical impact of anti-dsDNA antibody sub-specificities.

The cited Wikipedia statement is worryingly close to a warning Chalmer has formulated: “Biased under-reporting of research should be outlawed” (37). If we consider current views on a clinical impact of anti-dsDNA antibodies, we have to realize that aspects and data that oppose their status as a prototypical biomarker for SLE are clearly under-reported. [**Figure 2** **A**, discussed in (13)]. This is clear as deviant reports provide us with unmistakable data on anti-dsDNA antibodies in non-SLE conditions [**Figure 2** **A**, discussed in (13)]. In contrast, a categorically positive correlation of anti-dsDNA antibodies with SLE and lupus nephritis is reported. The SLE classification criterion - “The anti-dsDNA antibody” - in singular represents a group of unique individual antibodies against DNA structures and probably none of them are unique for SLE. This is discussed in detail below.

## DNA Structures and Anti-DNA Antibodies

In the modern history of DNA discoveries, different structures of DNA have been described (38, 39). Their unique roles are basically to facilitate and regulate DNA repair, replication and transcription of genes. Insight into these structures have provided us with basic understanding of genetics and DNA biology. Notably, the structures have a striking, yet largely overlooked relevance in an autoimmune context: Each structure has, aside from its basic function, a unique ability to induce highly specific anti-dsDNA antibodies (see below).

A central research focus has over decades been to describe elements of dsDNA and chromatin fragments as stimulators of B cells and T cells in context of SLE [(12, 24, 30, 40), discussed below, and in (13)]. Autoimmune hepatitis, for example, has recently become a focus in this context (41).

Studies of DNA and chromatin structures have indeed promoted fertile scientific achievements (see **Table 2** for a short history). Settled DNA/chromatin structures have provided us with insight into immunological processes that regulate tolerance for chromatin, but also into basic aspects of the immune system itself (see **Table 2**). Some research directions have, however, been hampered by deficient strategic hypotheses (see central problems described in **Table 1**).

**Table 2:** Central scientists and the resolution of the DNA structure.

**Miesher et al.** (42, 43) •Described in 1871 Nuclein later known as DNA. **Levene et al.** (44, 45) •Discovered ribose in 1909 and deoxyribose in 1929. •Suggested the structure of nucleic acids as a repeating nucleotide tetramer. •DNA contained adenine, guanine, thymine, cytosine, deoxyribose, phosphate group. **Chargaff et al.** (46–48) Defined in 1950, 1952 the 2 Chargaff rules •In double-stranded DNA, guanine units is equal to cytosine units, adenine units is equal to thymine units. •The composition of DNA varies between species. **Franklin et al.** (49–51) •In 1952 Franklin produced high-resolution photographs of crystallized DNA fibers, interpreted as a helical structure. She and Chargaff were close to defining the structure of DNA. •Franklin described the A and B forms of DNA. **Watson and Crick** (52, 53) •Used X-ray data from Franklin when they solved the helical structure of DNA in which A pairs with T, and C with G (equal to Chargaff´s 1. rule).

In light of this cognition, it is therefore a substandard statement to underline that modern clinical immunology and rheumatology propose that antibodies to dsDNA are a fundamental single unit and a central SLE classification criterium. This is stated in the “The 1982 revised American College of Rheumatology (ACR) SLE classification criteria”, in

“The Systemic Lupus International Collaborating Clinics (SLICC) classification criteria for systemic lupus erythematosus”, and in “The 2019 European League Against Rheumatism/American College of Rheumatology Classification Criteria for Systemic Lupus Erythematosus” (54–56) where the anti-dsDNA antibodies are defined irrespective structural DNA specificity. The focus on “The anti-dsDNA antibody” as a separate and specific criterion has most probably derailed a productive and critical, clinically relevant, research focus on anti-dsDNA antibodies. This relates to definition of them as a diagnostic marker as well as a pathogenic factor in SLE. This dilemma puts the critical focus on specificity versus avidity of these antibodies.

## Antibodies Against DNA Structures: Diversity of Specificities or Diversity of Avidities—Facts and Contrafacts

The next interpretative problem derived from the fact that “The anti-dsDNA antibody” bind differently in assay systems like enzyme-linked immuno-sorbent assay (ELISA), Crithidia luciliae immune-fluorescent test (CLIFT), the Farr and other assays [see e.g. (57, 58) and **Figure 2** **B**]. Binding in one or the other assay has been misinterpreted as if “The anti-dsDNA antibody” possesses a spectrum of avidities (59) – and not a spectrum of different unique DNA specificities as may appear in individual assay system, like binding of antibodies against bent B DNA as in core nucleosomes or in plasmid DNA (as in CLIFT), while antibodies to elongated linear B DNA, cruciform dsDNA, ssDNA in transcriptionally active chromatin, are all detected by e.g. ELISA assays using DNA designed for each structure, or to Z DNA formed in high salt as in the Farr assay (38, 60–62). It is relevant to stress that ssDNA in clinical immunology is often erroneously described as denatured DNA, and not as a real functional DNA structure (see below for details).

Thus, different antibodies have distinct specificities, and all autoimmune IgG anti-dsDNA antibodies produced *in vivo* are principally antigen-driven by any of the whole spectrum of DNA structures described in chromatin (38, 39). They are consequently affinity maturated and may all be of high avidity [see e.g. (11, 20, 21, 63, 64)]. Thus, specificity of antibodies for DNA structures may have informative impact on classification of SLE and on pathogenicity in SLE and lupus nephritis [discussed in (65)].

Importantly, this rationalization opens for a new understanding of the distinctions between specificity versus avidity and the consequent pathogenic impact of anti-DNA antibodies. Furthermore, this interpretation strongly supports the view that pathogenic impact of anti-dsDNA antibodies may encompass all possible anti-DNA antibody sub-specificities towards structures exposed in extra-cellular chromatin.

This (still mostly) theoretical discussion puts a focus on the nature, origin and function of individual antibodies recognizing dsDNA in all of its structural forms shaped in biologically active chromatin. This puts the focus on origin of these antibodies.

## Origin of Specific Anti-dsDNA Antibodies—A Concise Analysis

In the aftermath of description of autoimmunity to dsDNA in 1957 (5–8), the complexity of tolerance-regulation of DNA immunity has led to contemporary studies of the immunogenic impact of dsDNA as presented in NETs (31, 66), secondary necrotic cells SNECs (67), and microparticles [(68, 69) discussed in (16, 35) and below]. For central milestones important for our understanding of tolerance and immunity to DNA, see **Table 3**.

**Table 3:** Central scientists and milestone studies of anti-dsDNA antibodies.

Autoimmunity towards dsDNA were after 1957 centered around SLE (30, 209). Its relation to infections, as described in 1938-1939 was over time neglected. **Winkenwerder** et al. (4), **Sevag et al.** (2, 210), **Menzel et al.** (3) •They described in 1938-1939 antibodies to DNA in bacterial infections. •Their data challenge the dogma of anti-dsDNA antibodies as a central biomarker for SLE. **Ceppellini et al.** (5), **Robbins et al.** (7), **Miescher et al.** (6), **Seligmann et al.** (8) •Described in 1957 antibodies to DNA in SLE. •Their discovery formed later the basis for the dogma of anti-dsDNA antibodies as a central biomarker for SLE. **Sercarz et al.** (40, 86) •Proposed the hapten-carrier model for B and T cell cooperation in autoimmunity. This concept had a considerable impact on experimental studies on immunogenicity of DNA. **Tonegawa et al.** (17) •Described in 1983 somatic mutations in the N-terminal part of the variable region of an antibody as a mechanism for generation of antibody diversity. **Hood et al.** (18) •They proposed a model for variable region gene rearrangement mediated by proteins which recognize the same conserved sequences adjacent to both light and heavy chain immunoglobulin gene segments. •An immunoglobulin heavy chain variable region gene is generated from three segments of DNA: VH, D and JH. **Weigert et al.** (211–213) •He discovered a fundamental mechanism of B cell tolerance which he entitled *receptor editing.* •Weigert was the first to describe immunoglobulin variable region somatic hypermutation which is an adaptive mechanism to increase avidity, and to converge specificity towards the immunogen. **Stollar et al.** (10, 61, 70, 214) Central pioneer on •Immunogenic potential of DNA structures •immunochemical characterization of DNA and •genetical mapping of anti-DNA antibodies. **Schwartz et al.** (215) •Immunogenicity of DNA and anti-dsDNA antibodies, centralized around SLE **Isenberg et al.** (216, 217) •Clinical impact of anti-DNA antibodies, analyses of large SLE cohorts. **Tsokos et al.** (30, 209, 218) •Performed studies of cellular and molecular pathogenic processes of systemic lupus erythematosus (SLE). •Central in the field of molecular abnormalities of immunity in SLE. **Pisetsky et al.** (12, 219) Studies on the immunological properties of DNA as related to two main topics: •The induction of anti-DNA responses in systemic lupus erythematosus •the stimulation of innate immunity by bacterial DNA. **Reizis et al.** (16) •The Reizis group provide strong arguments for the view that DNA may represent an epicenter in SLE as B cell antigen and pathogenic factor as extra-cellular target for the anti-dsDNA antibodies. •They propose and provide data that DNase 1L3 prevents autoimmunity towards DNA. **Winkler et al.** (112) •Provided evidence that an affinity-maturated DNA specific autoantibody emerged from an antibody with undetectable affinity for DNA. The somatically mutated heavy chain variable region from the DNA-specific antibody was reverted by site-directed mutagenesis to germline configuration with loss of specificity for DNA. They made the important conclusion that affinity-maturated autoantibodies may develop during a normal immune response from non-autoimmune B cells. In light of high rates of infections their study may have high impact to understand origin of anti-dsDNA antibodies. This adds to data demonstrating that (nucleosomal) dsDNA also directly have immunogenic potential when complexed with an immunogenic carrier protein. **Other central contemporary scientists** (14, 152, 187, 220–224) •They have investigated origin, clinical and pathogenic impact of anti-DNA antibodies. They are all important and they are referred to in this study.

Regrettably, we have to admit that studies on autoimmunity to dsDNA have been less conclusive and thus less successful than the foregoing studies describing structure and function of the DNA helix and chromatin. Still, our insight into clinically relevant DNA-induced autoimmunity is founded on phenomenology, artificial experiments and hypothetical interpretations [discussed in (13)]. We can, however, argue for the view that DNA *structure-specific* antibodies are selectively induced by individual DNA configurations present in chromatin (**Figure 1**), and not merely by the vast number of cross-reactive non-DNA, non-polynucleotide structures [see detailed discussion below, and (13, 70)]. In light of this, “The anti-dsDNA antibody” is a critically inconsistent and erroneous term that does not open for further insight into the clinical impact of anti-DNA antibodies.

### Autoimmunity Versus Immunity of DNA—Two Roads Leading to the Same Center?

**Figure 3:**
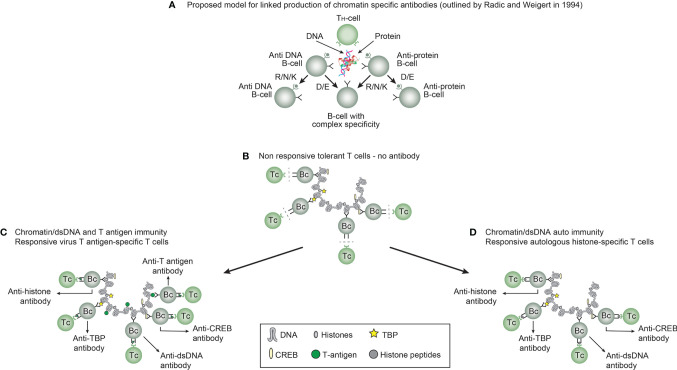
*Models for how production of anti-DNA antibodies may be initiated.* In order to understand results of experimental and empirical studies aimed to describe origin of anti-dsDNA antibodies, we need to settle a semantic distinction: Anti-dsDNA antibodies may be the result of immune responses to DNA-protein complexes in two different contexts: *immunity versus autoimmunity* **(A–D)**. A*utoimmunity* signifies an autoimmune response promoted solely by autologous dsDNA in complex with chromatin-derived autologous proteins. *Immunity* implies immune responses to dsDNA/chromatin in complex with a non-self (like infection-derived) DNA-binding protein component. In general, antibodies to dsDNA generated *in vivo* is most probably a result of both categories of immunity. There are many reasons to argue for the validity of these models to generate anti-DNA antibodies. These arguments were basically presented as *a theoretical model for the future* by Radic and Weigert in 1994 [presented in **(A)**]. In this model, aspects of affinity maturation is demonstrated as the B cell Ig variable regions are undergoing mutations to basic or acidic residues (This figure, Panel a, is re-drawn from a figure in reference (11), and provided by courtesy of Dr. Marko Radic, University of Tennessee Health Science Center). Deriving from this theoretical model, functional evidence-based models by Marion et al., Pisetsky, et al., and Rekvig et al. (32, 71–73) are demonstrated. In absence of responsive T cells a model for tolerance is presented **(B)**, and imply no help for DNA/chromatin-specific B cells. The distinction between immunity and autoimmunity is demonstrated in Panels c an d, respectively. The principle difference relies on the specificity of the T cells. In immunity, the T cells are specific for and engaged by non-self derived DNA-binding proteins **(C)**, while in autoimmunity, the T cells are engaged by autologous, chromatin-derived proteins like histones **(D)**. The basic model promoted by Radic and Weigert predicts a molecular and cellular prototype model also for linked production of antibodies to DNA, histones and other chromatin associated proteins, in accordance with theoretical reflections provided by Craft and Hardin already in 1987 (74). The repertoire of chromatin-specific autoantibodies is from theoretic consideration the same for the models presented in **(C, D)** (see text for details).

In order to probe hypotheses linked to experimental and empirical studies aimed to describe origin of anti-dsDNA antibodies, we need to settle a semantic distinction: Anti-dsDNA antibodies may be the result of immune responses to DNA-protein complexes in context of 2 principally different mechanisms for termination of tolerance: *Autoimmunity versus immunity* (see **Figure 3** **A–D**, for principle models).

This distinction is important to bring to the discussion forum; *autoimmunity* signifies an autoimmune response promoted solely by autologous dsDNA in complex with chromatin-derived autologous proteins, while *immunity* implies immune responses to dsDNA/chromatin in complex with a non-self (like infection-derived) DNA-binding protein component. In general, antibodies to dsDNA generated *in vivo* is most probably a result of both categories of immunity (concise models as described in **Figure 3** **C, D**, respectively).

There are many reasons to argue for the validity of these models to generate anti-DNA antibodies. These arguments were basically presented as *a theoretical model for the future* by Radic and Weigert in 1994 [presented in **Figure 3A** (11)] and as experimental evidence-based models by Marion et al., Pisetsky et al., and Rekvig et al. (32, 71–73). In absence of responsive T cells a model for tolerance is presented (**Figure 3** **B**), and imply no help for DNA/chromatin-specific B cells. **Figure 3** presents a basic model in scenarios linked to both immunity and autoimmunity of DNA (**Figure 3** **C, D**, respectively). The basic model promoted by Radic and Weigert predicts a molecular and cellular prototype model also for linked production of antibodies to DNA, histones and other chromatin associated proteins, in accordance with theoretical reflections provided by Craft and Hardin already in 1987 (74). The immunity model (**Figure 3** **C**) and the autoimmune model (**Figure 3** **D**) are validated by descriptive observations and experimental data [see thorough discussions in reference (12, 13)].

### Autoimmunity to dsDNA: An Autologous Origin of Key Proteins That Render dsDNA Immunogenic

**Figure 4:**
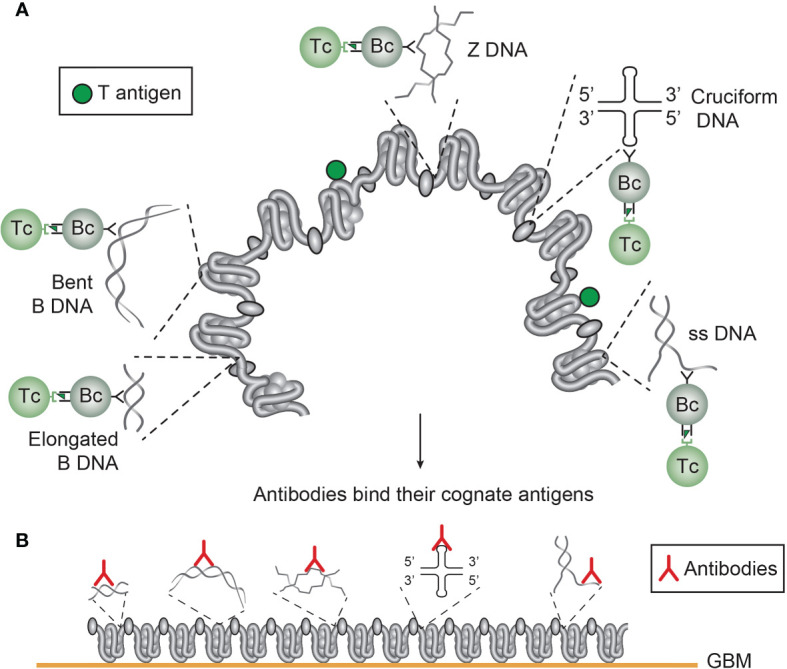
Several unique DNA structures are accessible for B cells that present immunogenic peptides from non-self DNA-binding proteins (here exemplified by polyomavirus T antigen). As indicated, all these exemplified structures are solvent phase and accessible to B cells **(A)**. In this figure, polyomavirus T antigen is associated with chromatin in infected cells, and all DNA-specific B cells that bind DNA/chromatin-T antigen complexes present T antigen peptides to responsive cognate T helper cells. The cognate interaction of DNA structure-specific B cells and T antigen peptide-specific T helper cells promote production of a repertoire of DNA structure-specific antibodies. Since these antigens are accessible to B cell antigen receptors, circulating antibodies may have access to, and bind, the same specter of antigens in chromatin exposed in e.g. glomerulus basement membranes **(B)**. This model emerges from published experimental data on immunogenicity of the selected DNA structures as is discussed in the present text. This model is also valid in a true autoimmune context. Responsive histone-specific T cells may fully substitute T antigen-specific T cells. This will allow the same specter of DNA structure-specific antibodies (see text for details).

**Figure 5:**
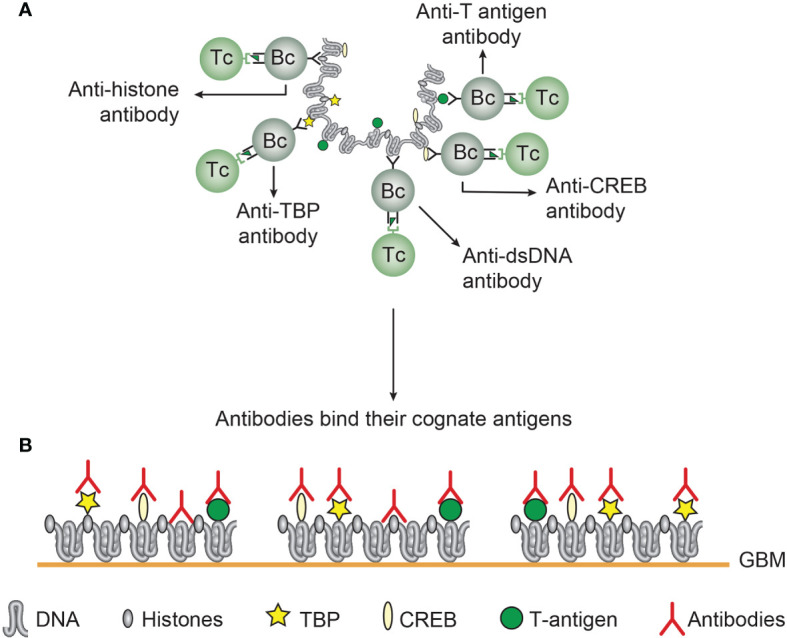
*Chromatin structures and immunogenic chromatin-associated proteins are accessible and may stimulate B and T cells.* In the figure all the selected molecules are accessible to B cells. As in **(A)**, polyomavirus T antigen is associated with chromatin in virus-infected cells, and all chromatin-specific B cells may present T antigen peptides to cognate T helper cells. This results in production of antibodies against unique DNA and chromatin/protein structures. Since these antigens are accessible to B cell antigen receptors, circulating antibodies may have the potential to bind the same specter of accessible antigens in chromatin exposed in e.g. glomerulus basement membranes **(B)**. This demonstrates that chromatin-specific antibodies per se may have pathogenic potentials, and not only anti-dsDNA antibodies. This model emerges from experimental data (see text). [This figure is modified from: **Figure 5** in reference (35)].

Still, we have not satisfactorily determined which molecular and cellular processes that are operational to promote production of anti-dsDNA antibodies *in vivo* (12, 13, 32, 75, 76), although DNA seems to be the B cell antigen (14, 16). A key question is why it is so difficult to experimentally induce anti-dsDNA antibodies *in vivo* without introducing non-self carrier proteins in complex with DNA/chromatin fragments. Examples are provided in **Figures 4** and **5**, for induction of anti-structural DNA antibodies and anti-chromatin antibodies, respectively by DNA/chromatin-polyomavirus T antigen complexes. This is consistent with high propensity to e.g. viral infections in SLE and cancers (71, 77, 78).

There are indeed studies that have demonstrated an immunogenic potential of purely autologous chromatin. Voll et al. demonstrated that histone-specific T cells (79), or release of chromatin-HMGB1 (High Mobility Group Box 1) complexes (80), promoted production of anti-dsDNA antibodies. HMGB1-containing nucleosomes from apoptotic cells were demonstrated to induce anti-dsDNA and anti-histone antibody responses, whereas nucleosomes taken from living cells did not (80).

Sisirak et al. (81) demonstrated that DNase 1L3 knock-out mice spontaneously produced anti-dsDNA antibodies and developed lupus nephritis. This study represented an experimental correlate to observations that an inherited null mutation of the DNase 1L3 gene is associated with early-onset SLE phenotype and development of lupus nephritis (82). In their study, Sisirak et al. made the logic conclusion that extracellular chromatin is a potential self-antigen normally digested by circulating DNase 1L3. This was further investigated by the Boris Reizis group, where the central effect on DNase 1L3 in prevention of autoimmunity towards DNA was ascertained, and that autoantibody-mediated inhibition of DNase1L3 activity facilitated anti-dsDNA autoreactivity in patients with severe sporadic SLE (81, 83). Restoration of DNase 1L3 activity could therefore represent a causal therapeutic approach to control the manifestations of SLE promoted by exposure of chromatin [discussed in (81, 83)]. Soni and Reizis provide strong arguments for the view that DNA may represent an epicenter in SLE as immunogen and pathogenic factor as extra-cellular target for the anti-dsDNA antibodies [(16), see also (31)].

The role of HMGB1- chromatin complexes to *promote* anti-DNA antibody production, and the role of DNase 1L3 to *prevent* production of anti-dsDNA antibodies represent important conceptual advantages in our search for understanding the molecular and cellular origin of anti-dsDNA antibodies.

### Immunity to dsDNA: An Infectious Origin of Key Proteins That Render dsDNA Immunogenic

Valid historical data argue for the view that anti-dsDNA antibodies are not unique for SLE (**Figure 2** **A**). Infections are commonly encountered in both SLE and in malignant diseases (73, 77, 78, 84, 85), a fact that may causally link anti-dsDNA antibodies to diseases prone to infections.

From studies of infectious-related anti-dsDNA antibody responses, we have insight into basic aspects of the molecular and cellular requirements to fulfill stimulation of the immune system [discussed in (11–13, 36, 70)]. One fairly well documented experimental model proclaims that DNA, as a hapten-like structure, a term introduced by Sercarz et al. (40, 86), must be complexed with certain immunogenic, *in vivo* expressed infection-derived DNA-binding carrier proteins [**Figure 3** **C** (27, 71, 75, 87)]. This has its experimental counterpart in using artificial carrier proteins like the widely used methylated bovine serum albumin [see e.g. (75, 87–89)].

In somewhat different, but important and thought-provoking studies, Pisetsky and co-workers investigated the autoimmunogenic properties of bacterial DNA (32, 90, 91). They took logic and important conceptual steps forwards by implementing CpG motifs as an additional (adjuvant-like) stimulation of the innate immune system (91–95).

### Autoimmunity to DNA: The Possible Role of Secondary Necrotic Cells (SNECs), Microparticles, or Neutrophil Extracellular Traps (NETs)

Alternatively, reduced clearance of apoptotic cells and consequently accumulation of SNECs (96, 97), microparticles (68), or exposure of NETs (31, 66, 98), have over time been attributed central roles in promotion of autoimmunity to native or to apoptosis-related modified chromatin-associated proteins and dsDNA possibly involved in e.g. lupus nephritis (99–102). However, no formal experimental evidences are presented that anti-dsDNA antibodies are *de facto* induced by SNECs or NETs. It is problematic that the relevant literature is categorized over decades as hypothesis and theories studies [see e.g. (16, 31, 66)], but not funded on solid reproducible experimental data. Such structures may, nevertheless, induce immunity towards proteins that are modified in e.g. NETs (in an altered self context), and may have central pathophysiological roles as targets for relevant autoantibodies (31).

### Cross-Stimulation of Anti-dsDNA Antibodies by Phospholipids, Peptides and Proteins

Non-DNA structures may promote production of anti-dsDNA antibodies [see e.g. (13, 65, 103–108)]. Such structures may encompass phospholipids that may share backbone structures similar to dsDNA (109), or peptides/protein structures with no apparent similarities to dsDNA (104, 108, 110, 111). A perfect example of evolution of anti-dsDNA antibodies that may have been promoted by a non-DNA structure is described by Wellman et al. (112). The IgG antibody with the heavy chain variable region in germline configuration did not bind DNA, while somatic mutations introduced during affinity maturation resulted in binding of the antibody to dsDNA.

However, critical questions must be raised in this context. No doubt that proteins or peptides may induce anti-dsDNA antibodies, but is this phenomenon exceptional? Have proteins and polypeptides the potential to induce antibodies against *all* different DNA structures and affinity maturate and converge specificity of the antibodies towards the manifold of DNA structures? Except for the Wellman-study, no published data yet provide answers to these questions.

These observations and discussions have till now not precipitated any conclusive evidence-based conclusions, although strong arguments can be raised that in sum support homologous stimulation of the immune system by native dsDNA in complex with a T cell-specific immunogenic carrier protein as a central trajectory *in vivo* (13).

## The Biology of Unique Forms of DNA and Their Immunogenicity *In Vivo*

This discussion is based on the contributions provided by historically important scientific pioneers, their observations and consequent interpretations: Characterization of dsDNA and subsequent description of antibodies to dsDNA. A common thread leads from the revolutionary discoveries of DNA and its structures by Miescher et al. in 1871 (42), Levene et al. in 1903 (44), Chargaff et al. in 1950 (46), Franklin et al. in 1953 (49), Watson and Crick in 1953 (52, 53), up to studies of DNA´s structure and function in chromatin by groups of Olins and Olins (113), Kornberg (114, 115), Klug (116), Laskey (117), and others. They contributed to our understanding of structure and biology of the symbiosis of dsDNA and chromatin-associated regulatory proteins (see **Table 2**).

Franklin was the first to describe unique forms of DNA beyond its pure helix structure: The A and B DNA (49). The B DNA was later described as a dynamic bi-structural DNA shape: elongated (118, 119) or bent (120) B DNA, while ssDNA (121, 122), Z DNA (61), cruciform DNA (123), and other structures were all described in context of specific functions of DNA (see detailed discussion below, **Figure 1**, and (124, 125).

In the history of “The anti-dsDNA antibody” few random attempts have been performed to determine whether all structural DNA-specificities are strongly associated with SLE. Thus, it is not established if unique anti-dsDNA antibody specificities are linked to distinct SLE classification criteria or even to non-SLE related disorders (13). These obvious problems are not considered in the recent expansion of the SLE classification criteria (54–56). In the following sections the roles of DNA structures, functions and their cognate antibodies will be summarized and discussed.

## B DNA

### Structure and Biology of B DNA

B DNA is the most disseminated DNA structure in the human genome. The fundamental composition (47, 48) and structure of the B form DNA as a right-handed double helix (49, 50, 126) reflect in many ways the basic B DNA in its relaxed low energy conformation. Changes in the B DNA structure reflect dynamic conversion of the basic structure into variants like ssDNA, Z DNA, cruciform DNA, bent DNA and others (see **Figures 1** and **4**). Such activation-related structures have their own, unique ability to induce highly specific immune responses, with relevance to the impact of anti-dsDNA in SLE and lupus nephritis.

The B DNA is reversibly transformed into two different B DNA structures with impact on specific immune responses: The elongated linker B DNA and the bent B DNA formed in the core nucleosome (for other structures, see below).

### Elongated B DNA

Linker DNA is a stretched elongated B DNA. Its name defines its context, a link between core nucleosomes, shaping the electron microscopy picture of beads on a string (113). The histone H1 binds to linker DNA where DNA connects the fundamental chromatin units, the core nucleosomes. The role of histone H1 in chromatin is manifold, and H1 contributes to chromatin compaction (127). H1 is a central molecule that basically unmask DNA and contribute to regulation of transcription and other effects involving DNA (128, 129). Thus, H1 is highly mobile in the nucleus, which may indicate its strategic ability to expose B DNA to DNA regulatory proteins.

### Bent dsDNA

As H1 slides along linker DNA, the histone octamer (two copies each of the four core histones H2A, H2B, H3 and H4) slides along B DNA and form bent DNA (120, 130–132), thus facilitating further effects of regulatory proteins like high-mobility group proteins to bend DNA into various degrees of flexible conformations (133–135). Studies on kinetoplast DNA [a network of circular DNA (136)] have demonstrated that certain sequences cause DNA to be highly bent, and that other sequences bend in response to binding of proteins (137). Thus, bent B DNA is a widely spread structure in chromatin, which may impact its immunogenic power.

B DNA structures undergo transformation between elongated and bent B DNA necessary to promote transcription and replication (132, 138, 139). The amount of bent DNA is therefore substantial (132). Summarizing this information, functional alterations of the DNA structure generate unique stimulators of the adaptive immune system in substantial amounts.

### Spontaneous Production of Anti-B DNA Antibodies

The origin and clinical impact of anti-B DNA antibodies (termed anti-mammalian dsDNA antibodies in clinical and immunological contexts) have been difficult to understand. Therefore, immune responses towards B DNA has been, and is still being regarded as enigmatic. The reason for this derives from two problems: DNA immunogenicity, and affinity maturation of ongoing immune responses against peptides or phospholipids mimicking or apparently not mimicking DNA although stimulating to anti-B DNA antibody production.

In many lupus-prone murine models [for review, see e.g. (140, 141)], antibodies against B DNA appear spontaneously. They distinctively recognize elongated and/or bent dsDNA as in chromatin and kinetoplasts in different assay systems (57). These spontaneously produced antibodies are pathogenic (but not always)! by promoting lupus nephritis (65), dermatitis (142) and some forms of cerebral lupus (143, 144). The capacity of some anti-DNA antibodies to promote inflammation in the kidneys is more rigorously documented than in the skin or brain. The reason for this is the documented devastating effect exerted by the organ-selective silencing of the renal endonuclease DNase 1. This leads to a consequent accumulation of extra-cellular large chromatin fragments in glomerular matrices and membranes where they are targeted by anti-dsDNA antibodies [(65) see below]. 345-352

### Experimental Production of Anti-B DNA Antibodies

Over the years, attempts to induce anti-B DNA antibodies have mostly failed [see e.g. (61, 76), reviewed in (13)]. Anti-DNA antibodies have been induced by other DNA structures like chemically modified DNA and synthetic polydeoxyribo-nucleotides that differ from native DNA [discussed in (70)]. After a period where B DNA was regarded as non-immunogenic, clear exceptions from these negative results have appeared.

The current contemporary view is that experimental induction of anti-B DNA operates according to mechanisms described above linked to autoimmunity or immunity against mammalian dsDNA. The early experiments were performed using new hapten-carrier principles: To engage T helper cells, a DNA binding peptide, Fus 1derived from Trypanosoma cruzii, induced in complex with mammalian B DNA strong anti-B DNA antibodies in non-autoimmune mice (14, 145). Immunoglobulin analyses demonstrated that the IgG heavy chain variable regions were structurally similar to those produced spontaneously in autoimmune (NZBxNZW)F1 mice (63, 145). An analogous approach was independently developed using the DNA-binding polyomavirus BK large T antigen as carrier protein for the hapten-analogous B DNA. The *in vivo* generation of this hapten-carrier complex promoted production of lupus-like autoantibodies to mammalian dsDNA and to chromatin-associated proteins [(73, 84), and references herein]. Experimental induction of antibodies recognizing the kinetoplast DNA of Crithidia luciliae along with elongated B DNA in ELISA were observed in both the Fus 1-DNA and the T antigen-DNA models.

The T antigen model was principally confirmed in another experimental system. Dong et al. induced antibodies to p53 by immunizing non-autoimmune mice with purified p53-T antigen complex (146). These results demonstrate that infections, commonly encountered in SLE (32) and in cancers (77, 78) may be involved in systemic autoimmunity, and explain why anti-dsDNA antibodies principally cannot serve as a unique biomarker for SLE.

### Pathogenic Impact of Anti-B DNA Antibodies

There is an international consensus that antibodies to dsDNA and to chromatin antigens have pathogenic potentials. There is, however, no consensus as to how and why these antibodies may be pathogenic (65). Two main directions in international science dominate the discussions: *i.* In context of lupus nephritis, antibodies bind chromatin fragments exposed in the mesangial matrix and in GBM [(147–150), discussed in (65)], or *ii.* Antibodies bind inherent matrix and GBM structures through cross-reactions [discussed in (151, 152)]. Antibodies against chromatin ligands *and* intrinsic glomerular constituents have been eluted from nephritic kidneys (153, 154). The main problem with those studies is that each of them claim to explain the nephritic potential of anti-dsDNA antibodies [discussed in depth in (33, 65)]. These contradictory results have not promoted critical, comparative studies. Before such studies are performed and interpreted, we will not reach consensus on which model(s) is (are) correct and which strategy for causal therapy may be developed (principally discussed below).

## ssDNA

### Structure and Biology of ssDNA

The ssDNA structure appears in two different contexts: *i.* as intended/not-intended denatured ssDNA in analytical contexts, or *ii.* related to stabilize transcriptionally active DNA (121, 122).

The ssDNA structure is not stable. Single-stranded DNA-binding proteins (SSB) hold the ssDNA intact and exposed during the course of its function: DNA transcription, recombination and repair (155), and to serve as template for opposite strand DNA synthesis [(156), for further reading, see e.g. (157, 158)]. Thus, ssDNA regions may be present in total cellular DNA at considerable amount, which may point at an immunogenic impact of ssDNA and a pathogenic impact of anti-ssDNA antibodies.

### Immunity of ssDNA Regions

Anti-ssDNA antibodies may be induced *in vivo* when functional chromatin-associated ssDNA is presented to the immune system (13). Therefore, anti-ssDNA antibodies may bind ssDNA regions in chromatin fragments also when they are exposed in e.g. GBM and thereby promote renal antibody-mediated inflammatory events. This is substantiated, but not seriously considered, by the fact that anti-ssDNA antibodies can be detected in sera and renal eluates from SLE patients with lupus nephritis (153, 159, 160).

### Spontaneous and Experimental Production of Anti-ssDNA Antibodies and Their Pathogenic Impact

Autoantibodies against ssDNA has been known for decades (13). They have been detected in SLE and other conditions (161), and they are readily induced experimentally (162–166). In one study from 1989, Vaishnav and Antony injected ssDNA in complex with a carrier protein (mBSA) and observed, as the first ever, appearance of anti-dsDNA antibodies (163). This study was at that time important and challenged the concept of non-immunogenicity of DNA including B DNA, but was not considered important. In later studies and discussions their results were regrettably neglected.

In my training, I was stressed to treat DNA as target in anti-dsDNA antibody assays with S1 nuclease to avoid detection of anti-ssDNA antibodies in clinical contexts (167). Therefore, antibodies against ssDNA regions have been disregarded in clinical contexts, although they have been detected in nephritic kidneys (153). Thus, also anti-ssDNA antibodies may affinity maturate and form high avidity antibodies with potential to promote lupus nephritis and dermatitis when chromatin fragments are exposed in situ.

## Z DNA

### Structure and Biology of Z-DNA

Z DNA is structurally and functionally integrated in the human genome (168) and is involved in various human diseases (see (169, 170) and references therein). Z-DNA is a left-handed, high energy supercoiled double helix, as opposed to the right-handed B-DNA helix. Physiologically, Z DNA forms *in vivo* and in cell cultures during transcription (171) as a result of torsional strain that depends on interaction of mobile polymerases and other proteins (172, 173). Since the placement of nucleosomes influences the binding of transcription factors, Z-DNA is thought to directly regulate the rate of transcription.

Z-DNA is reported to be formed in elongated B DNA and not associated with the core nucleosome unit, which are normally located after Z-DNA structures (174). Concerns have, however, been expressed by Mulholland et al. (175), who have demonstrated that Z DNA may also be formed in the core nucleosomal complex. This indicates that Z DNA may be more abundant in chromatin with an increased probability for immunogenicity and a pathogenic potential of anti-Z DNA antibodies. A pathogenic potential of anti-Z DNA antibodies has not been proven by firm descriptive or experimental studies.

### Spontaneous Production of Anti-Z DNA Antibodies

Specific anti-Z-DNA antibodies are associated with SLE (176, 177). Significant amounts of anti Z-DNA antibodies were found in SLE patients but not in other rheumatic diseases – analyses in infections or malignancies are, however, not reported. Highest levels of antibodies were associated with the most active stages of SLE.

### Experimentally Induced Anti-Z DNA Antibodies

In the period when immunogenicity of DNA was a major problematic focus in clinically related immunology, B DNA was regarded as non-immunogenic (76, 178). In contrast, anti-Z DNA antibodies were readily induced by conventional immunization protocols (87, 179). These contrasting results were insightfully discussed (61), and subsequent experiments revealed that mBSA as carrier protein was functional for Z DNA, but not for B DNA although other carrier proteins had the potential to render B DNA immunogenic [see e.g. (14, 180)].

### Anti-Z DNA Antibodies: Potential Pathogenic Impact

IgG antibodies to Z DNA are found in SLE, and they have been experimentally induced in non-autoimmune mice (see above). This confers to affinity maturated antibodies with potentially high avidity. When we consider the fact that Z DNA is involved in transcription and recombination, Z DNA may be abundantly exposed in chromatin, and also in chromatin fragments released and exposed *in situ* in e.g. GBM. This may open for a pathogenic potential for these antibodies (see a theoretical model discussed in **Figure 4**). If anti-Z DNA antibodies indeed are pathogenic has, however, not been investigated.

## Cruciform dsDNA

### Structure and Biology of Cruciform DNA

Cruciform DNA is structurally different from B and Z DNA. Its formation requires that inverted sequences (palindromes) present in one strand is repeated on the other strand in opposite direction, thus allowing formation of hairpin or cruciform DNA structures. There is a minimum limit of the number of nucleotides in the inverted repeats to form a stable cruciform structure by negative DNA supercoiling. The cruciform structures are, like Z DNA, higher energy DNA structures [for details see (123, 181)].

Cruciform DNA structures are central in a wide range of biological processes, including replication, regulation of gene expression, nucleosome structure and recombination. Several regulatory proteins bind preferentially, but not exclusively to cruciform structures, and regulate homeostasis of the biological functions of DNA (123, 182, 183).

### Spontaneously Produced Anti-Cruciform DNA Autoantibodies: Still Not Analyzed

Cruciform DNA-specific antibodies have not been reported in a clinical context, and no attempts to detect these antibodies in context of rheumatology or infectivity have been published.

### Experimental Anti-Cruciform DNA Antibodies

Antibodies were induced experimentally in 1987 by Frappier et al. against a cruciform structure presented by a heteroduplex DNA molecule (184). Their well characterized monoclonal antibodies have later been used to study expression and biology of cruciform DNA (182, 185). Notably, also antibodies towards another complex form of DNA, quadruplex DNA, was generated from non-immunized motheaten mice (182, 185). This may indicate that anti-cruciform/anti-quadruplex antibodies may be formed in autoimmune phenotypes., although not investigated yet.

### Possible Pathogenic Impact of Anti-Cruciform Autoantibodies—A Hypothesis

Pathogenic impact of anti-cruciform DNA antibodies has not been investigated. This is a consequence of the fact that there are no published reports on true autoimmune anti-cruciform antibodies linked to autoimmune diseases. However, when we consider the central functions of cruciform DNA in biology, and that cruciform DNA structures are abundant in chromatin, these structures are expected to be recognized by the cognate immune system.

## Unique DNA Structures as Stimulators and Targets for Antibodies—A Conclusion

In this theoretical study, available information linked to immune responses to various structural forms of DNA is contemplated and interpreted: *i.* the role of infection in initiation of anti-DNA production, *ii.* the possible influence of microbiota that turns out to be unbalanced in lupus (186), *iii.* the molecular and structural properties of ssDNA/dsDNA in chromatin and their interaction with B cells (afferent immunogenic stimulus) and anti-DNA antibodies (efferent pathogenic stimulus) (12, 13).

DNA as a native structure is immunogenic and auto-immunogenic *in vivo*. The emerging antibodies do not care what initiates them but their existence is undeniable. Their clinical impact is, however, tremendous. In this picture chromatin exposed *in situ* is a common denominator as target for the whole specter of induced anti-DNA/anti-chromatin antibodies. Cross-reactions with membrane ligands play assumedly an inferior pathogenic role, because it is unlikely that the whole universe of DNA/chromatin-specific autoantibodies cross-react with the small repertoire of protein ligands that make up matrices and membranes.

## Which Anti-chromatin Antibodies Are Nephritogenic—A Hypothesis

In this section anti-DNA antibodies as principal initiators of lupus nephritis will be discussed. Secondary inflammatory mediators and processes will not be emphasized here. Anti-dsDNA antibodies (in the contexts discussed above) are among several anti-chromatin antibodies involved in lupus nephritis. We still, however, do not agree on the nature of inducers and glomerular targets of the anti-dsDNA antibodies - whether dsDNA (11, 149, 150, 187), nucleosomes or apoptotic chromatin (147, 148, 188–190) or non-dsDNA cross-reactive structures [see e.g. (13, 65, 103–108)]. This signifies that we today are not able to explain the nephritic process. We can, however, deduce some basic principles and propose some data-based paradigms.

If autoantibodies bind directly to intrinsic ligands in the matrix or GBM, then this mode is equivalent to a Type II antibody-dependent inflammation. If autoantibodies form immune complexes with chromatin fragments *in situ* or in circulation prior to deposition in e.g. GBM, this mode is consistent with a Type III immune complex-mediated inflammation [for review, see (191)].

The chromatin model is complex, and involve a spectrum of chromatin-specific antibodies as indicated in **Figures 4** and **5**. Immunogenic chromatin stimulates production of different anti-chromatin antibodies. The model in **Figure 4** informs about how immunogenic DNA structures may stimulate production of cognate DNA-specific anti-DNA autoantibodies. On the other hand, immunogenic chromatin has the potential to promote production of a spectrum of anti-chromatin antibodies, like DNA, histones, transcription factors ((84, 180), **Figure 5**). Collectively. these antibodies have not been seriously considered as individual promoters of lupus nephritis, with the exception of “The anti-dsDNA antibody”. Since all these antibodies, with the exception of anti-cruciform antibodies (not analyzed yet), are induced in SLE (57, 58, 176), the DNA structures must have been accessible to B cells. Then it is likely that the antibodies recognize the same universe of DNA structures and chromatin-associated proteins (**Figures 4** and **5**) when chromatin fragments are exposed in e.g. glomeruli.

Although the spectrum of chromatin autoantibodies may bind chromatin in situ, this does not necessarily imply that each specificity is individually nephritogenic since the density of each target molecule may be too low to initiate e.g. complement activation. However, they may all contribute to the nephritogenic process in concert with other chromatin-specific antibodies. This hypothesis is consistent with the fact that non-anti-DNA IgG antibodies are eluted from lupus nephritic kidneys [(153), discussed in (153, 159, 160)].

This process is also consistent with previous data demonstrating that *in vivo*-bound IgG antibodies co-localize with electron-dense chromatin fragments in the mesangial matrix and in GBM [discussed in (65, 148, 188, 192, 193)]. In addition, antibodies in glomerular eluates demonstrated higher intrinsic affinity for DNA compared to autologous serum antibodies (194).

Data that emerge from these analyses were not consistent with antibody-binding to membrane constituents, as e.g. laminin antibodies added to the sections bound normal GBM and not electron-dense chromatin fragments [see **Figure 3** in reference (65)]. Collectively, these data favor a Type III inflammatory model involved in lupus nephritis, although autoimmune T cells may also be involved (195).

The arguments favoring Type II and Type III nephritis derive from studies over decades, preliminary conclusions and from a lack of international consensus (see e.g. contrasting viewpoints in (13, 33, 34, 65, 149, 196). The two models have their advocates, but still a comparative study is awaited.

### Serologically Active, Clinically Quiescent Patients—Why Are Not Anti-DNA Antibodies Always Pathogenic?

This question relates to the statement that describes “serologically active, clinically quiescent” patients (197, 198). The term describes patients that have long-lasting high levels of anti-DNA antibodies without experiencing any inflammatory flare of their disease. This is in fact a core problem aimed to understand the complexity of the pathogenic impact of anti-DNA antibodies: When and how is the anti-dsDNA antibody pathogenic?

Two explanations may allow an understanding of this apparent paradox. Either, the antibodies do not possess an *a priori* nephritogenic potential just because of their presence. This implies that the target(s) for the anti-DNA antibodies is not constitutively expressed and exposed *in vivo*, i.e. they are not an intrinsic part of e.g. GBM. Only in situations where e.g. chromatin accumulate extra-cellularly, the antibodies find their partner and upon binding promote inflammation (188, 199, 200). The loss of DNase 1 endonuclease activity in kidneys but not in other organs (201) may also explain why kidneys are more affected by anti-DNA antibodies as DNase 1 deficiency promotes glomerular exposure of chromatin fragments (202).

The alternative explanation could be that the antibodies account for inflammation if they cross-react with intrinsic membrane constituents like laminin, collagen or entactin. Without cross-reactive potential the anti-DNA antibodies behave as a clinical epiphenomenon [see above, discussed in detail in (13, 65)]. These two models have one perspective in common: They both provide a fair explanation as to why anti-DNA antibodies are not always pathogenic and why patients may be “serologically active, clinically quiescent”. A comparative research initiative to solve the real process is an important challenge to us.

### Pathogenicity of Anti-DNA Antibodies—Does Immunoglobulin Class Matter?

A potentially important pathogenic aspect of anti-DNA antibodies adheres to the impact of their immunoglobulin class (203). Although IgM antibodies possess low intrinsic affinity their avidity is generally high. This, and the fact that a single IgM molecule is a potent complement activator (204, 205), whereas single IgG molecule hardly activate complement (206), could indicate that IgM anti-DNA antibodies are more pathogenic than IgG. The opposite seems to be true (203). In their study, Wang and Xia conclude that IgG but not IgM correlate with activity of human lupus nephritis (203). Most pathogenic antibodies are of the IgG class in SLE patients (207). According to e.g. Gronwall et al., IgM antibodies correlated with enhanced removal of apoptotic material and reduced activity of lupus erythematosus (208). These observations implicate that IgG anti-dsDNA antibodies exert a stronger pathogenic impact than IgM antibodies with corresponding DNA specificity.

## Concluding Remarks

In current criteria to classify SLE, “The anti-dsDNA antibody” possesses an archaic position. “The anti-dsDNA antibody”-terminology is neither founded on current knowledge, nor on established insight into unique DNA structures related to distinct DNA-associated operations. *In that sense, any structure-specific anti-dsDNA antibody, detected in any assay using any DNA molecule is valid as a criterium for SLE.* A simple hypothesis - not examined yet - may be quite obvious: The more readily an antibody is induced, the less specific is the antibody for SLE, but may appear in divergent conditions. In other words, antibodies against ssDNA or Z DNA may be less specific for SLE than anti-bent B DNA (extrapolated from data discussed above and in **Figure 2**).

Anti-DNA antibodies are essential in clinical medicine, and particularly in SLE. The autoantibodies are, although as an interim measure, used to diagnose SLE and to classify SLE patients. The antibodies are a central pathogenic factor, and they promote lupus nephritis alone or in combination with other anti-chromatin antibodies. What we need to comprehend from this enormous amount of data and knowledge is to understand what makes the anti-DNA antibody pathogenic - and in which context.

## Ethics Statement

The present manuscript is a review on murine and human SLE and lupus nephritis. All data are taken from original studies approved by relevant ethical committees.

## Author Contributions

The author confirms being the sole contributor of this work and has approved it for publication.

## Conflict of Interest

The author declares that the research was conducted in the absence of any commercial or financial relationships that could be construed as a potential conflict of interest.

## Publisher’s Note

All claims expressed in this article are solely those of the authors and do not necessarily represent those of their affiliated organizations, or those of the publisher, the editors and the reviewers. Any product that may be evaluated in this article, or claim that may be made by its manufacturer, is not guaranteed or endorsed by the publisher.

## Footnotes

1 References listed in this section are meant as valid examples, and do not intend to represent a complete biography of aspects of anti-dsDNA antibodies.

2
**Ludwik Fleck** (1896 –1961) was a Polish physician, biologist and philosopher. In the 1930s he developed the philosophical concepts of "*Denkstil*" ("thought style") and the "*Denkkollektiv*" ("thought collective"). Fleck´s concept of the "thought collective" is central in the philosophy of science and in logology (the "science of science") and was a leading force in the Polish school of logology. He used his consept to describe non-linear evolution of scientific ideas in a “thought collective”, much as in Thomas Kuhn´s "periodic paradigm shift" concept.

3 Per August 2021 more than 41.700 publications are found in PubMed using the term “anti-DNA antibodies”

4 https://en.wikipedia.org/wiki/Anti-dsDNA_antibodies

## References

[1] Fleck L. Entstehung Und Entwicklung Einer Wissenschaftlichen Tatsache. In: Einführung in Die Lehre Vom Denkstil Und Denkkollektiv. Frankfurt am Main: Suhrkamp (1980).

[2] Sevag MG Lackman DB Smolen J. The Isolation of the Components of Streptococcal Nucleoproteins in Serologically Active Form. J Biol Chem (1938) 124:425–36. doi: 10.1016/S0021-9258(18)74048-9

[3] Menzel AEO Heidelberger M. Cell Protein Fractions of Bovine and Avian Tubercle Bacillus Strains and of the Timothy-Grass Bacillus. J Biol Chem (1938) 124:301–7. doi: 10.1016/S0021-9258(18)74098-2

[4] Winkenwerder WL Buell MV Howard JE. The Sensitizing Properties of the Nucleic Acids and Their Derivatives. Science (1939) 90:356. doi: 10.1126/science.90.2337.356 17843027

[5] Ceppellini R Polli E Celada F. A DNA-Reacting Factor in Serum of a Patient With Lupus Erythematosus Diffusus. Proc Soc Exp Biol Med (1957) 96:572–4. doi: 10.3181/00379727-96-23544 13505795

[6] Miescher P Strassle R. New Serological Methods for the Detection of the L.E. Factor. Vox Sang (1957) 2(4):283–7. doi: 10.1111/j.14230410.1957.tb03704.x 13496698

[7] Robbins WC Holman HR Deicher H Kunkel HG. Complement Fixation With Cell Nuclei and DNA in Lupus Erythematosus. Proc Soc Exp Biol Med (1957) 96:575–9. doi: 10.3181/00379727-96-23545 13505796

[8] Seligmann M. Demonstration in the Blood of Patients With Disseminated Lupus Erythematosus a Substance Determining a Precipitation Reaction With Desoxyribonucleic Acid. C R Hebd Seances Acad Sci (1957) 245(2):243–5. 13461391

[9] Califf RM. Biomarker Definitions and Their Applications. Exp Biol Med (Maywood ) (2018) 243(3):213–21. doi: 10.1177/1535370217750088 PMC5813875 29405771

[10] Stollar BD. The Biochemistry and Genetics of DNA and Anti-DNA Antibodies. Clin Rheumatol (1990) 9(1 Suppl 1):30–8. doi: 10.1007/BF02205549 2203593

[11] Radic MZ Weigert M. Genetic and Structural Evidence for Antigen Selection of Anti- DNA Antibodies. Annu Rev Immunol (1994) 12:487–520. doi: 10.1146/annurev.iy.12.040194.002415 8011289

[12] Pisetsky DS. Anti-DNA Antibodies - Quintessential Biomarkers of SLE. Nat Rev Rheumatol (2016) 12(2):102–10. doi: 10.1038/nrrheum.2015.151 26581343

[13] Rekvig OP. The Anti-DNA Antibody: Origin and Impact, Dogmas and Controversies. Nat Rev Rheumatol (2015) 11:530–40. doi: 10.1038/nrrheum.2015.69 26034836

[14] Desai DD Krishnan MR Swindle JT Marion TN. Antigen-Specific Induction of Antibodies Against Native Mammalian DNA in Nonautoimmune Mice. J Immunol (1993) 151(3):1614–26. 8393048

[15] Chen C Prak EL Weigert M. Editing Disease-Associated Autoantibodies. Immunity (1997) 6(1):97–105. doi: 10.1016/s1074-7613(00)80673-1 9052841

[16] Soni C Reizis B. Self-DNA at the Epicenter of SLE: Immunogenic Forms, Regulation, and Effects. Front Immunol (2019) 10:1601. doi: 10.3389/fimmu.2019.01601 31354738 PMC6637313

[17] Tonegawa S. Somatic Generation of Antibody Diversity. Nature (1983) 302(5909):575–81. doi: 10.1038/302575a0 6300689

[18] Early P Huang H Davis M Calame K Hood L. An Immunoglobulin Heavy Chain Variable Region Gene Is Generated From Three Segments of DNA: VH, D and JH. Cell (1980) 19(4):981–92. doi: 10.1016/0092-8674(80)90089-6 6769593

[19] Li Z Woo CJ Iglesias-Ussel MD Ronai D Scharff MD. The Generation of Antibody Diversity Through Somatic Hypermutation and Class Switch Recombination. Genes Dev (2004) 18(1):1–11. doi: 10.1101/gad.1161904 14724175

[20] Shlomchik M Mascelli M Shan H Radic MZ Pisetsky D Marshak Rothstein A. Anti-DNA Antibodies From Autoimmune Mice Arise by Clonal Expansion and Somatic Mutation. J Exp Med (1990) 171(1):265–92. doi: 10.1084/jem.171.1.265 PMC2187662 2104919

[21] Krishnan MR Marion TN. Comparison of the Frequencies of Arginines in Heavy Chain CDR3 of Antibodies Expressed in the Primary B-Cell Repertoires of Autoimmune-Prone and Normal Mice. Scand J Immunol (1998) 48(3):223–32. doi: 10.1046/j.1365-3083.1998.00426.x 9743205

[22] Shuster AM Gololobov GV Kvashuk OA Bogomolova AE Smirnov IV Gabibov AG. DNA Hydrolyzing Autoantibodies. Science (1992) 256(5057):665–7. doi: 10.1126/science.1585181 1585181

[23] Kostrikina IA Buneva VN Nevinsky GA. Systemic Lupus Erythematosus: Molecular Cloning of Fourteen Recombinant DNase Monoclonal Kappa Light Chains With Different Catalytic Properties. Biochim Biophys Acta (2014) 1840(6):1725–37. doi: 10.1016/j.bbagen.2014.01.027 24462577

[24] Stollar BD. Immunochemical Analyses of Nucleic Acids. Prog Nucleic Acid Res Mol Biol (1992) 42:39–77. doi: 10.1016/s0079-6603(08)60573-5 1574590

[25] Qiu CC Caricchio R Gallucci S. Triggers of Autoimmunity: The Role of Bacterial Infections in the Extracellular Exposure of Lupus Nuclear Autoantigens. Front Immunol (2019) 10:2608. doi: 10.3389/fimmu.2019.02608 31781110 PMC6857005

[26] Khan SN Witsch EJ Goodman NG Panigrahi AK Chen C Jiang Y. Editing and Escape From Editing in Anti-DNA B Cells. Proc Natl Acad Sci USA (2008) 105(10):3861–6. doi: 10.1073/pnas.0800025105 PMC2268806 18310318

[27] Cerutti ML Zarebski LM de Prat GG Goldbaum FA. A Viral DNA-Binding Domain Elicits Anti-DNA Antibodies of Different Specificities. Mol Immunol (2005) 42(3):327–33. doi: 10.1016/j.molimm.2004.09.003 15589321

[28] Madrid FF Maroun MC Olivero OA Long M Stark A Grossman LI. Autoantibodies in Breast Cancer Sera Are Not Epiphenomena and may Participate in Carcinogenesis. BMC Cancer (2015) 15:407. doi: 10.1186/s12885-015-1385-8 25975273 PMC4453436

[29] Franks AL Slansky JE. Multiple Associations Between a Broad Spectrum of Autoimmune Diseases, Chronic Inflammatory Diseases and Cancer. Anticancer Res (2012) 32(4):1119–36. PMC3349285 22493341

[30] Tsokos GC. Autoimmunity and Organ Damage in Systemic Lupus Erythematosus. Nat Immunol (2020) 21(6):605–14. doi: 10.1038/s41590-020-0677-6 PMC8135909 32367037

[31] Pieterse E van d V. Breaking Immunological Tolerance in Systemic Lupus Erythematosus. Front Immunol (2014) 5:164. doi: 10.3389/fimmu.2014.00164 24782867 PMC3988363

[32] Pisetsky DS Vrabie IA. Antibodies to DNA: Infection or Genetics? Lupus (2009) 18(13):1176–80. doi: 10.1177/0961203309106492 19880564

[33] Goilav B Putterman C. The Role of Anti-DNA Antibodies in the Development of Lupus Nephritis: A Complementary, or Alternative, Viewpoint? Semin Nephrol (2015) 35(5):439–43. doi: 10.1016/j.semnephrol.2015.08.005 PMC4662078 26573546

[34] van Bavel CC Fenton KA Rekvig OP van d V Berden JH. Glomerular Targets of Nephritogenic Autoantibodies in Systemic Lupus Erythematosus. Arthritis Rheum (2008) 58(7):1892–9. doi: 10.1002/art.23626 18576314

[35] Rekvig OP. Systemic Lupus Erythematosus: Definitions, Contexts, Conflicts, Enigmas. Front Immunol (2018) 9:387. doi: 10.3389/fimmu.2018.00387 29545801 PMC5839091

[36] Rekvig OP. Anti-dsDNA Antibodies as a Classification Criterion and a Diagnostic Marker for Systemic Lupus Erythematosus: Critical Remarks. Clin Exp Immunol (2015) 179(1):5–10. doi: 10.1111/cei.12296 24533624 PMC4260890

[37] Chalmers I. Biased Underreporting of Research Is Unethical and Should Be Outlawed. Z Arztl Fortbild Qualitatssich (2006) 100(7):531–5. 17137067

[38] Kaushik M Kaushik S Roy K Singh A Mahendru S Kumar M. A Bouquet of DNA Structures: Emerging Diversity. Biochem Biophys Rep (2016) 5:388–95. doi: 10.1016/j.bbrep.2016.01.013 PMC5600441 28955846

[39] van Steensel B. Chromatin: Constructing the Big Picture. EMBO J (2011) 30(10):1885–95. doi: 10.1038/emboj.2011.135 PMC3098493 21527910

[40] Ando DG Sercarz EE Hahn BH. Mechanisms of T and B Cell Collaboration in the *In Vitro* Production of Anti-DNA Antibodies in the NZB/NZW F1 Murine SLE Model. J Immunol (1987) 138(10):3185–90. 2952711

[41] Granito A Muratori L Tovoli F Muratori P. Diagnostic Role of anti-dsDNA Antibodies: Do Not Forget Autoimmune Hepatitis. Nat Rev Rheumatol (2021) 17(4):244. doi: 10.1038/s41584-021-00573-7 33462415

[42] Miescher F. Ueber Die Chemische Zusammensetzung Der Eiterzellen. Medizinisch-Chemische Untersuchungen (1871) 4:441–60.

[43] Dahm R. Friedrich Miescher and the Discovery of DNA. Dev Biol (2005) 278(2):274–88. doi: 10.1016/j.ydbio.2004.11.028 15680349

[44] Levene PA. On the Chemistry of the Chromatin Substance of the Nerve Cell. J Med Res (1903) 10(2):204–11. PMC2105936 19971569

[45] Frixione E Ruiz-Zamarripa L. The "Scientific Catastrophe" in Nucleic Acids Research That Boosted Molecular Biology. J Biol Chem (2019) 294(7):2249–55. doi: 10.1074/jbc.CL119.007397 PMC6378961 30765511

[46] Chargaff E Magasanik B Vischer E Green C Doniger R Elson D. Nucleotide Composition of Pentose Nucleic Acids From Yeast and Mammalian Tissues. J Biol Chem (1950) 186(1):51–67. 14778803

[47] Chargaff E Zamenhof S Green C. Composition of Human Desoxypentose Nucleic Acid. Nature (1950) 165(4202):756–7. doi: 10.1038/165756b0 15416834

[48] Elson D Chargaff E. On the Desoxyribonucleic Acid Content of Sea Urchin Gametes. Experientia (1952) 8(4):143–5. doi: 10.1007/BF02170221 14945441

[49] Franklin RE Gosling RG. Evidence for 2-Chain Helix in Crystalline Structure of Sodium Deoxyribonucleate. Nature (1953) 172(4369):156–7. doi: 10.1038/172156a0 13072614

[50] Maddox B. Rosalind Franklin: The Dark Lady of DNA. Serial (Book,Monograph: HarperCollins. Ref Type (1957).

[51] Elkin LO. Rosalind Franklin and the Double Helix. Phys Today (2003) 56(3):42. doi: 10.1063/1.1570771

[52] Watson JD Crick FH. The Structure of DNA. Cold Spring Harb Symp Quant Biol (1953) 18:123–31. doi: 10.1101/sqb.1953.018.01.020 13168976

[53] Watson JD Crick FH. Genetical Implications of the Structure of Deoxyribonucleic Acid. Nature (1953) 171(4361):964–7. doi: 10.1038/171964b0 13063483

[54] Tan EM Cohen AS Fries JF Masi AT McShane DJ Rothfield NF. The 1982 Revised Criteria for the Classification of Systemic Lupus Erythematosus. Arthritis Rheum (1982) 25(11):1271–7. doi: 10.1002/art.1780251101 7138600

[55] Petri M Orbai AM Alarcon GS Gordon C Merrill JT Fortin PR. Derivation and Validation of the Systemic Lupus International Collaborating Clinics Classification Criteria for Systemic Lupus Erythematosus. Arthritis Rheum (2012) 64(8):2677–86. doi: 10.1002/art.34473 PMC3409311 22553077

[56] Aringer M Costenbader K Daikh D Brinks R Mosca M Ramsey-Goldman R. 2019 European League Against Rheumatism/American College of Rheumatology Classification Criteria for Systemic Lupus Erythematosus. Arthritis Rheumatol (2019) 71(9):1400–12. doi: 10.1002/art.40930 PMC6827566 31385462

[57] Haugbro K Nossent JC Winkler T Figenschau Y Rekvig OP. Anti-dsDNA Antibodies and Disease Classification in Antinuclear Antibody Positive Patients: The Role of Analytical Diversity. Ann Rheum Dis (2004) 63(4):386–94. doi: 10.1136/ard.2003.016303 PMC1754943 15020332

[58] Compagno M Rekvig OP Bengtsson AA Sturfelt G Heegaard NH Jonsen A. Clinical Phenotype Associations With Various Types of anti-dsDNA Antibodies in Patients With Recent Onset of Rheumatic Symptoms. Results From a Multicentre Observational Study. Lupus Sci Med (2014) 1(1):e000007. doi: 10.1136/lupus-2013-000007 25396058 PMC4225731

[59] Smeenk R van der Lelij G Aarden L. Avidity of Antibodies to dsDNA: Comparison of IFT on Crithidia Luciliae, Farr Assay, and PEG Assay. J Immunol (1982) 128(1):73–8. 7033379

[60] Kim SH Lim SH Lee AR Kwon DH Song HK Lee JH. Unveiling the Pathway to Z-DNA in the Protein-Induced B-Z Transition. Nucleic Acids Res (2018) 46(8):4129–37. doi: 10.1093/nar/gky200 PMC5934635 29584891

[61] Stollar BD. Why the Difference Between B-DNA and Z-DNA? Lupus (1997) 6(3):327–8. doi: 10.1177/096120339700600327 9296781

[62] Lafer EM Sousa R Ali R Rich A Stollar BD. The Effect of Anti-Z-DNA Antibodies on the B-DNA-Z-DNA Equilibrium. J Biol Chem (1986) 261(14):6438–43. doi: 10.1016/S0021-9258(19)84581-7 3700399

[63] Marion TN Tillman DM Krishnan MK Desai DD Jou NT Ruff MB. Immunoglobulin Variable-Region Structures in Immunity and Autoimmunity to DNA. Tohoku J Exp Med (1994) 173(1):43–63. doi: 10.1620/tjem.173.43 7809911

[64] Gilkeson GS Bernstein K Pippen AM Clarke SH Marion T Pisetsky DS. The Influence of Variable-Region Somatic Mutations on the Specificity and Pathogenicity of Murine Monoclonal Anti-DNA Antibodies. Clin Immunol Immunopathol (1995) 76(1 Pt 1):59–67. doi: 10.1006/clin.1995.1088 7606869

[65] Rekvig OP. The dsDNA, Anti-dsDNA Antibody, and Lupus Nephritis: What We Agree on, What Must Be Done, and What the Best Strategy Forward Could be. Front Immunol (2019) 10:1104. doi: 10.3389/fimmu.2019.01104 31156647 PMC6529578

[66] Dwived N Marko R. Burning Controversies in NETs and Autoimmunity: The Mysteries of Cell Death and Autoimmune Disease. Autoimmunity (2018) 51(6):267–80. doi: 10.1080/08916934.2018.1523395 30417698

[67] Biermann MHC Boeltz S Pieterse E Knopf J Rech J Bilyy R. Autoantibodies Recognizing Secondary NEcrotic Cells Promote Neutrophilic Phagocytosis and Identify Patients With Systemic Lupus Erythematosus. Front Immunol (2018) 9:989. doi: 10.3389/fimmu.2018.00989 29867966 PMC5949357

[68] Mobarrez F Svenungsson E Pisetsky DS. Microparticles as Autoantigens in Systemic Lupus Erythematosus. Eur J Clin Invest (2018) 48(12):e13010. doi: 10.1111/eci.13010 30062774

[69] Beyer C Pisetsky DS. The Role of Microparticles in the Pathogenesis of Rheumatic Diseases. Nat Rev Rheumatol (2010) 6(1):21–9. doi: 10.1038/nrrheum.2009.229 19949432

[70] Stollar BD. Antibodies to DNA. CRC Crit Rev Biochem (1986) 20(1):1–36. doi: 10.3109/10409238609115899 3514122

[71] Van Ghelue M Moens U Bendiksen S Rekvig OP. Autoimmunity to Nucleosomes Related to Viral Infection: A Focus on Hapten-Carrier Complex Formation. J Autoimmun (2003) 20(2):171–82. doi: 10.1016/s0896-8411(02)00110-5 12657530

[72] Kohm AP Fuller KG Miller SD. Mimicking the Way to Autoimmunity: An Evolving Theory of Sequence and Structural Homology. Trends Microbiol (2003) 11(3):101–5. doi: 10.1016/s0966-842x(03)00006-4 12648936

[73] Rekvig OP Bendiksen S Moens U. Immunity and Autoimmunity Induced by Polyomaviruses: Clinical, Experimental and Theoretical Aspects. Adv Exp Med Biol (2006) 577:117–47. doi: 10.1007/0-387-32957-9_9 16626032

[74] Craft JE Hardin JA. Linked Sets of Antinuclear Antibodies: What do They Mean? J Rheumatol Suppl (1987) 14 Suppl 13:106–9. 2441041

[75] Stollar BD. Immunochemistry of DNA. Int Rev Immunol (1989) 5(1):1–22. doi: 10.3109/08830188909086987 2491157

[76] Madaio MP Hodder S Schwartz RS Stollar BD. Responsiveness of Autoimmune and Normal Mice to Nucleic Acid Antigens. J Immunol (1984) 132(2):872–6. 6690621

[77] Zur HH. Viruses in Human Cancers. Science (1991) 254(5035):1167–73. doi: 10.1126/science.1659743 1659743

[78] Zur HH. The Search for Infectious Causes of Human Cancers: Where and Why (Nobel Lecture). Angew Chem Int Ed Engl (2009) 48(32):5798–808. doi: 10.1002/anie.200901917 19588476

[79] Voll RE Roth EA Girkontaite I Fehr H Herrmann M Lorenz HM. Histone-Specific Th0 and Th1 Clones Derived From Systemic Lupus Erythematosus Patients Induce Double-Stranded DNA Antibody Production. Arthritis Rheum (1997) 40(12):2162–71. doi: 10.1002/art.1780401210 9416853

[80] Urbonaviciute V Furnrohr BG Meister S Munoz L Heyder P De MF. Induction of Inflammatory and Immune Responses by HMGB1-Nucleosome Complexes: Implications for the Pathogenesis of SLE. J Exp Med (2008) 205(13):3007–18. doi: 10.1084/jem.20081165 PMC2605236 19064698

[81] Sisirak V Sally B D’Agati V Martinez-Ortiz W Ozcakar ZB David J. Digestion of Chromatin in Apoptotic Cell Microparticles Prevents Autoimmunity. Cell (2016) 166(1):88–101. doi: 10.1016/j.cell.2016.05.034 27293190 PMC5030815

[82] Al-Mayouf SM Sunker A Abdwani R Abrawi SA Almurshedi F Alhashmi N. Loss-Of-Function Variant in DNASE1L3 Causes a Familial Form of Systemic Lupus Erythematosus. Nat Genet (2011) 43(12):1186–8. doi: 10.1038/ng.975 22019780

[83] Hartl J Serpas L Wang Y Rashidfarrokhi A Perez OA Sally B. Autoantibody-Mediated Impairment of DNASE1L3 Activity in Sporadic Systemic Lupus Erythematosus. J Exp Med (2021) 218(5). doi: 10.1084/jem.20201138 PMC8020718 33783474

[84] Moens U Seternes OM Hey AW Silsand Y Traavik T Johansen B. *In Vivo* Expression of a Single Viral DNA-Binding Protein Generates Systemic Lupus Erythematosus-Related Autoimmunity to Double-Stranded DNA and Histones. Proc Natl Acad Sci USA (1995) 92(26):12393–7. doi: 10.1073/pnas.92.26.12393 PMC40364 8618908

[85] Jacqueline C Tasiemski A Sorci G Ujvari B Maachi F Misse D. Infections and Cancer: The "Fifty Shades of Immunity" Hypothesis. BMC Cancer (2017) 17(1):257. doi: 10.1186/s12885-017-3234-4 28403812 PMC5389015

[86] Eardley DD Sercarz EE. Modulation of Help and Suppression in a Hapten-Carrier System. J Immunol (1976) 116(3):600–5. 56397

[87] Edgington SM Stollar BD. Immunogenicity of Z-DNA Depends on the Size of Polynucleotide Presented in Complexes With Methylated BSA. Mol Immunol (1992) 29(5):609–17. doi: 10.1016/0161-5890(92)90197-6 1584229

[88] Gilkeson GS Ruiz P Howell D Lefkowith JB Pisetsky DS. Induction of Immune-Mediated Glomerulonephritis in Normal Mice Immunized With Bacterial DNA. Clin Immunol Immunopathol (1993) 68(3):283–92. doi: 10.1006/clin.1993.1129 8370182

[89] Gilkeson GS Pippen AM Pisetsky DS. Induction of Cross-Reactive anti-dsDNA Antibodies in Preautoimmune NZB/NZW Mice by Immunization With Bacterial DNA. J Clin Invest (1995) 95(3):1398–402. doi: 10.1172/JCI117793 PMC441482 7883986

[90] Pisetsky DS Grudier JP Gilkeson GS. A Role for Immunogenic DNA in the Pathogenesis of Systemic Lupus Erythematosus. Arthritis Rheum (1990) 33(2):153–9. doi: 10.1002/art.1780330202 2407246

[91] Jiang W Pisetsky DS. Enhancing Immunogenicity by CpG DNA. Curr Opin Mol Ther (2003) 5(2):180–5. 12772509

[92] Hanagata N. Structure-Dependent Immunostimulatory Effect of CpG Oligodeoxynucleotides and Their Delivery System. Int J Nanomed (2012) 7:2181–95. doi: 10.2147/IJN.S30197 PMC3356174 22619554

[93] Pisetsky DS. The Role of Innate Immunity in the Induction of Autoimmunity. Autoimmun Rev (2008) 8(1):69–72. doi: 10.1016/j.autrev.2008.07.028 18708168

[94] Krieg AM Vollmer J. Toll-Like Receptors 7, 8, and 9: Linking Innate Immunity to Autoimmunity. Immunol Rev (2007) 220:251–69. doi: 10.1111/j.1600-065X.2007.00572.x 17979852

[95] Krieg AM. The CpG Motif: Implications for Clinical Immunology. BioDrugs (1998) 10(5):341–6. doi: 10.2165/00063030-199810050-00001 18020606

[96] Gaipl US Kuhn A Sheriff A Munoz LE Franz S Voll RE. Clearance of Apoptotic Cells in Human SLE. Curr Dir Autoimmun (2006) 9:173–87. doi: 10.1159/000090781 16394661

[97] Kruse K Janko C Urbonaviciute V Mierke CT Winkler TH Voll RE. Inefficient Clearance of Dying Cells in Patients With SLE: anti-dsDNA Autoantibodies, MFG-E8, HMGB-1 and Other Players. Apoptosis (2010) 15:1098–1113. doi: 10.1007/s10495-010-0478-8 20198437

[98] Munoz LE Kaplan MJ Radic M Herrmann M. Editorial: NETosis 2: The Excitement Continues. Front Immunol (2017) 8:1318. doi: 10.3389/fimmu.2017.01318 29104572 PMC5655467

[99] Dieker J Berden JH Bakker M Briand JP Muller S Voll R. Autoantibodies Against Modified Histone Peptides in SLE Patients Are Associated With Disease Activity and Lupus Nephritis. PloS One (2016) 11(10):e0165373. doi: 10.1371/journal.pone.0165373 27780265 PMC5079581

[100] Karlsen AE Dyrberg T. Molecular Mimicry Between Non-Self, Modified Self and Self in Autoimmunity. Semin Immunol (1998) 10(1):25–34. doi: 10.1006/smim.1997.0102 9529653

[101] Dalum I Jensen MR Gregorius K Thomasen CM Elsner HI Mouritsen S. Induct of Cross-Reactive Antibodies Against a Self Protein by Immunization With a Modified Self Protein Containing a Foreign T Helper Epitope. Mol Immunol (1997) 34(16-17):1113–20. doi: 10.1016/s0161-5890(97)00147-8 9566759

[102] Rother N Pieterse E Lubbers J Hilbrands L van d V. Acetylated Histones in Apoptotic Microparticles Drive the Formation of Neutrophil Extracellular Traps in Active Lupus Nephritis. Front Immunol (2017) 8:1136. doi: 10.3389/fimmu.2017.01136 28959262 PMC5604071

[103] Mostoslavsky G Fischel R Yachimovich N Yarkoni Y Rosenmann E Monestier M. Lupus Anti-DNA Autoantibodies Cross-React With a Glomerular Structural Protein: A Case for Tissue Injury by Molecular Mimicry. Eur J Immunol (2001) 31(4):1221–7. doi: 10.1002/1521-4141(200104)31:4<1221::aid-immu1221>3.0.co;2-p 11298348

[104] Deocharan B Zhou Z Antar K Siconolfi-Baez L Angeletti RH Hardin J. Alpha-Actinin Immunization Elicits Anti-Chromatin Autoimmunity in Nonautoimmune Mice. J Immunol (2007) 179(2):1313–21. doi: 10.4049/jimmunol.179.2.1313 17617624

[105] Zhang W Reichlin M. A Possible Link Between Infection With Burkholderia Bacteria and Systemic Lupus Erythematosus Based on Epitope Mimicry. Clin Dev Immunol (2008) 2008:683489. doi: 10.1155/2008/683489 18682819 PMC2494591

[106] Qureshi F Yang Y Jaques SM Johnson MP Naparstek Y Ulmansky R. Anti-DNA Antibodies Cross-Reacting With Laminin Inhibit Trophoblast Attachment and Migration: Implications for Recurrent Pregnancy Loss in SLE Patients. Am J Reprod Immunol (2000) 44(3):136–42. doi: 10.1111/j.8755-8920.2000.440302.x 11028899

[107] Yadav P Carr MT Yu R Mumbey-Wafula A Spatz LA. Mapping an Epitope in EBNA-1 That Is Recognized by Monoclonal Antibodies to EBNA-1 That Cross-React With dsDNA. Immun Inflammation Dis (2016) 4(3):362–75. doi: 10.1002/iid3.119 PMC5004290 27621818

[108] Beger E Deocharan B Edelman M Erblich B Gu Y Putterman C. A Peptide DNA Surrogate Accelerates Autoimmune Manifestations and Nephritis in Lupus-Prone Mice. J Immunol (2002) 168(7):3617–26. doi: 10.4049/jimmunol.168.7.3617 11907127

[109] Lafer EM Rauch J Andrzejewski C Jr. Mudd D Furie B Furie B. Polyspecific Monoclonal Lupus Autoantibodies Reactive With Both Polynucleotides and Phospholipids. J Exp Med (1981) 153(4):897–909. doi: 10.1084/jem.153.4.897 6972993 PMC2186121

[110] Putterman C Diamond B. Immunization With a Peptide Surrogate for Double-Stranded DNA (dsDNA) Induces Autoantibody Production and Renal Immunoglobulin Deposition. J Exp Med (1998) 188(1):29–38. doi: 10.1084/jem.188.1.29 9653081 PMC2525538

[111] Ray SK Putterman C Diamond B. Pathogenic Autoantibodies Are Routinely Generated During the Response to Foreign Antigen: A Paradigm for Autoimmune Disease. Proc Natl Acad Sci USA (1996) 93(5):2019–24. doi: 10.1073/pnas.93.5.2019 PMC39902 8700878

[112] Wellmann U Letz M Herrmann M Angermuller S Kalden JR Winkler TH. The Evolution of Human Anti-Double-Stranded DNA Autoantibodies. Proc Natl Acad Sci USA (2005) 102(26):9258–63. doi: 10.1073/pnas.0500132102 PMC1166593 15968001

[113] Olins AL Olins DE. Spheroid Chromatin Units (V Bodies). Science (1974) 183(4122):330–2. doi: 10.1126/science.183.4122.330 4128918

[114] Kornberg RD Lorch Y. Chromatin-Modifying and -Remodeling Complexes. Curr Opin Genet Dev (1999) 9(2):148–51. doi: 10.1016/s0959-437x(99)80022-7 10322131

[115] Kornberg RD Lorch Y. Twenty-Five Years of the Nucleosome, Fundamental Particle of the Eukaryote Chromosome. Cell (1999) 98(3):285–94. doi: 10.1016/s0092-8674(00)81958-3 10458604

[116] Richmond TJ Finch JT Rushton B Rhodes D Klug A. Structure of the Nucleosome Core Particle at 7 A Resolution. Nature (1984) 311(5986):532–7. doi: 10.1038/311532a0 6482966

[117] Laskey RA Mills AD Philpott A Leno GH Dilworth SM Dingwall C. The Role of Nucleoplasmin in Chromatin Assembly and Disassembly. Philos Trans R Soc Lond B Biol Sci (1993) 339(1289):263–9. doi: 10.1098/rstb.1993.0024 8098530

[118] Bosaeus N Reymer A Beke-Somfai T Brown T Takahashi M Wittung-Stafshede P. A Stretched Conformation of DNA With a Biological Role? Q Rev Biophys (2017) 50:e11. doi: 10.1017/S0033583517000099 29233223

[119] Veloso A Kirkconnell KS Magnuson B Biewen B Paulsen MT Wilson TE. Rate of Elongation by RNA Polymerase II Is Associated With Specific Gene Features and Epigenetic Modifications. Genome Res (2014) 24(6):896–905. doi: 10.1101/gr.171405.113 24714810 PMC4032854

[120] Caraglio M Skoruppa E Carlon E. Overtwisting Induces Polygonal Shapes in Bent DNA. J Chem Phys (2019) 150(13):135101. doi: 10.1063/1.5084950 30954045

[121] Ashton NW Bolderson E Cubeddu L O’Byrne KJ Richard DJ. Human Single-Stranded DNA Binding Proteins Are Essential for Maintaining Genomic Stability. BMC Mol Biol (2013) 14:9. doi: 10.1186/1471-2199-14-9 23548139 PMC3626794

[122] Richard DJ Bolderson E Khanna KK. Multiple Human Single-Stranded DNA Binding Proteins Function in Genome Maintenance: Structural, Biochemical and Functional Analysis. Crit Rev Biochem Mol Biol (2009) 44(2-3):98–116. doi: 10.1080/10409230902849180 19367476

[123] Brazda V Laister RC Jagelska EB Arrowsmith C. Cruciform Structures Are a Common DNA Feature Important for Regulating Biological Processes. BMC Mol Biol (2011) 12:33. doi: 10.1186/1471-2199-12-33 21816114 PMC3176155

[124] Ghosh A Bansal M. A Glossary of DNA Structures From A to Z. Acta Crystallogr D Biol Crystallogr (2003) 59:620–6. doi: 10.1107/s0907444903003251 12657780

[125] Dickerson RE. DNA Structure From A to Z. Methods Enzymol (1992) 211:67–111. doi: 10.1016/0076-6879(92)11007-6 1406328

[126] Watson JD Crick FH. A Structure for Deoxyribose Nucleic Acid. 1953 Nat (2003) 421(6921):397–8. 12569935

[127] Kowalski A Palyga J. Modulation of Chromatin Function Through Linker Histone H1 Variants. Biol Cell (2016) 108(12):339–56. doi: 10.1111/boc.201600007 27412812

[128] Izzo A Schneider R. The Role of Linker Histone H1 Modifications in the Regulation of Gene Expression and Chromatin Dynamics. Biochim Biophys Acta (2016) 1859(3):486–95. doi: 10.1016/j.bbagrm.2015.09.003 26348411

[129] Fyodorov DV Zhou BR Skoultchi AI Bai Y. Emerging Roles of Linker Histones in Regulating Chromatin Structure and Function. Nat Rev Mol Cell Biol (2018) 19(3):192–206. doi: 10.1038/nrm.2017.94 29018282 PMC5897046

[130] Armeev GA Kniazeva AS Komarova GA Kirpichnikov MP Shaytan AK. Histone Dynamics Mediate DNA Unwrapping and Sliding in Nucleosomes. Nat Commun (2021) 12(1):2387. doi: 10.1038/s41467-021-22636-9 33888707 PMC8062685

[131] Wu C Travers A. Relative Affinities of DNA Sequences for the Histone Octamer Depend Strongly Upon Both the Temperature and Octamer Concentration. Biochemistry (2005) 44(43):14329–34. doi: 10.1021/bi050915w 16245949

[132] Richmond TJ Davey CA. The Structure of DNA in the Nucleosome Core. Nature (2003) 423(6936):145–50. doi: 10.1038/nature01595 12736678

[133] Grosschedl R Giese K Pagel J. HMG Domain Proteins: Architectural Elements in the Assembly of Nucleoprotein Structures. Trends Genet (1994) 10(3):94–100. doi: 10.1016/0168-9525(94)90232-1 8178371

[134] Murugesapillai D McCauley MJ Maher LJ III Williams MC. Single-Molecule Studies of High-Mobility Group B Architectural DNA Bending Proteins. Biophys Rev (2017) 9(1):17–40. doi: 10.1007/s12551-016-0236-4 28303166 PMC5331113

[135] Drozdetski AV Mukhopadhyay A Onufriev AV. Strongly Bent Double-Stranded DNA: Reconciling Theory and Experiment. Front Phys (2019) 7:195. doi: 10.3389/fphy.2019.00195 32601596 PMC7323118

[136] Shlomai J. The Structure and Replication of Kinetoplast DNA. Curr Mol Med (2004) 4(6):623–47. doi: 10.2174/1566524043360096 15357213

[137] Rosanio G Widom J Uhlenbeck OC. *In Vitro* Selection of DNAs With an Increased Propensity to Form Small Circles. Biopolymers (2015) 103(6):303–20. doi: 10.1002/bip.22608 25620396

[138] Schleif R. DNA Looping. Annu Rev Biochem (1992) 61:199–223. doi: 10.1146/annurev.bi.61.070192.001215 1497310

[139] Luger K Richmond TJ. DNA Binding Within the Nucleosome Core. Curr Opin Struct Biol (1998) 8(1):33–40. doi: 10.1016/s0959-440x(98)80007-9 9519294

[140] Perry D Sang A Yin Y Zheng YY Morel L. Murine Models of Systemic Lupus Erythematosus. J BioMed Biotechnol (2011) 2011:271694. doi: 10.1155/2011/271694 21403825 PMC3042628

[141] Richard ML Gilkeson G. Mouse Models of Lupus: What They Tell Us and What They Don’t. Lupus Sci Med (2018) 5(1):e000199. doi: 10.1136/lupus-2016-000199 29387435 PMC5786947

[142] Fismen S Hedberg A Fenton K Jacobsen S Krarup E Kamper A. Circulating Chromatin-Anti-Chromatin Antibody Complexes Bind With High Affinity to Dermo-Epidermal Structures in Murine and Human Lupus Nephritis. Lupus (2009) 18(7):597–607. doi: 10.1177/0961203308100512 19433459

[143] DeGiorgio LA Konstantinov KN Lee SC Hardin JA Volpe BT Diamond B. A Subset of Lupus Anti-DNA Antibodies Cross-Reacts With the NR2 Glutamate Receptor in Systemic Lupus Erythematosus. Nat Med (2001) 7(11):1189–93. doi: 10.1038/nm1101-1189 11689882

[144] Huerta PT Kowal C DeGiorgio LA Volpe BT Diamond B. Immunity and Behavior: Antibodies Alter Emotion. Proc Natl Acad Sci USA (2006) 103(3):678–83. doi: 10.1073/pnas.0510055103 PMC1334673 16407105

[145] Krishnan MR Marion TN. Structural Similarity of Antibody Variable Regions From Immune and Autoimmune Anti-DNA Antibodies. J Immunol (1993) 150(11):4948–57. 8496596

[146] Dong X Hamilton KJ Satoh M Wang J Reeves WH. Initiation of Autoimmunity to the P53 Tumor Suppressor Protein by Complexes of P53 and SV40 Large T Antigen. J Exp Med (1994) 179(4):1243–52. doi: 10.1084/jem.179.4.1243 PMC2191430 8145041

[147] Kalaaji M Fenton KA Mortensen ES Olsen R Sturfelt G Alm P. Glomerular Apoptotic Nucleosomes Are Central Target Structures for Nephritogenic Antibodies in Human SLE Nephritis. Kidney Int (2007) 71(7):664–72. doi: 10.1038/sj.ki.5002133 17332738

[148] Kalaaji M Mortensen E Jorgensen L Olsen R Rekvig OP. Nephritogenic Lupus Antibodies Recognize Glomerular Basement Membrane-Associated Chromatin Fragments Released From Apoptotic Intraglomerular Cells. Am J Pathol (2006) 168(6):1779–92. doi: 10.2353/ajpath.2006.051329 PMC1606630 16723695

[149] Berden JH Licht R Van Bruggen MC Tax WJ. Role of Nucleosomes for Induction and Glomerular Binding of Autoantibodies in Lupus Nephritis. Curr Opin Nephrol Hypertens (1999) 8(3):299–306. doi: 10.1097/00041552-199905000-00005 10456260

[150] van d V Berden JH. Lupus Nephritis: Role of Antinucleosome Autoantibodies. Semin Nephrol (2011) 31(4):376–89. doi: 10.1016/j.semnephrol.2011.06.009 21839371

[151] van Bavel CC van d V Berden JH. Glomerular Binding of anti-dsDNA Autoantibodies: The Dispute Resolved? Kidney Int (2007) 71(7):600–1. doi: 10.1038/sj.ki.5002126 17387307

[152] Krishnan MR Wang C Marion TN. Anti-DNA Autoantibodies Initiate Experimental Lupus Nephritis by Binding Directly to the Glomerular Basement Membrane in Mice. Kidney Int (2012) 82(2):184–92. doi: 10.1038/ki.2011.484 PMC3343188 22297676

[153] Xie C Liang Z Chang S Mohan C. Use of a Novel Elution Regimen Reveals the Dominance of Polyreactive Antinuclear Autoantibodies in Lupus Kidneys. Arthritis Rheum (2003) 48(8):2343–52. doi: 10.1002/art.11092 12905490

[154] Winfield JB Faiferman I Koffler D. Avidity of Anti-DNA Antibodies in Serum and IgG Glomerular Eluates From Patients With Systemic Lupus Erythematosus. Association of High Avidity Antinative DNA Antibody With Glomerulonephritis. J Clin Invest (1977) 59(1):90–6. doi: 10.1172/JCI108626 PMC333335 299748

[155] Marceau AH. Functions of Single-Strand DNA-Binding Proteins in DNA Replication, Recombination, and Repair. Methods Mol Biol (2012) 922:1–21. doi: 10.1007/978-1-62703-032-8_1 22976174

[156] Yuanzhong Wu JLTK. Human Single-Stranded DNA Binding Proteins: Guardians of Genome Stability. Acta Biochim Biophys Sin (2016) 48(7):671–7. doi: 10.1093/abbs/gmw044 27217471

[157] Pfeifer M Brem R Lippert TP Boulianne B Ho HN Robinson ME. SSB1/SSB2 Proteins Safeguard B Cell Development by Protecting the Genomes of B Cell Precursors. J Immunol (2019) 202(12):3423–33. doi: 10.4049/jimmunol.1801618 PMC6545462 31085591

[158] Pandita RK Chow TT Udayakumar D Bain AL Cubeddu L Hunt CR. Single-Strand DNA-Binding Protein SSB1 Facilitates TERT Recruitment to Telomeres and Maintains Telomere G-Overhangs. Cancer Res (2015) 75(5):858–69. doi: 10.1158/0008-5472.CAN-14-2289 PMC4351820 25589350

[159] Sabbaga J Pankewycz OG Lufft V Schwartz RS Madaio MP. Cross-Reactivity Distinguishes Serum and Nephritogenic Anti-DNA Antibodies in Human Lupus From Their Natural Counterparts in Normal Serum. J Autoimmun (1990) 3(2):215–35. doi: 10.1016/0896-8411(90)90142-f 2340059

[160] Mannik M. Use of Novel Elution Regimens of Autoantibodies in Lupus Kidneys: Comment on the Article by Xie Et al. Arthritis Rheum (2004) 50(5):1699–700. doi: 10.1002/art.20329 15146451

[161] Pavlovic M Kats A Cavallo M Chen R Hartmann JX Shoenfeld Y. Pathogenic and Epiphenomenal Anti-DNA Antibodies in SLE. Autoimmune Dis (2010) 2011:462841. doi: 10.4061/2010/462841 21152217 PMC2989704

[162] Starobinski M Lacour M Reininger L Izui S. Autoantibody Repertoire Analysis in Normal and Lupus-Prone Mice. J Autoimmun (1989) 2(5):657–74. doi: 10.1016/s0896-8411(89)80005-8 2803476

[163] Vaishnav YN Antony A. Antibodies Raised Against Denatured DNA Bind to Double-Stranded DNA. J Immunol Methods (1989) 118(1):25–30. doi: 10.1016/0022-1759(89)90048-3 2926149

[164] Pancer LB Bell DA Singhal SK. Induction of anti-ssDNA Antibodies in Normal and Preautoimmune Mice In Vivo. J Immunol (1980) 124(2):939–46. 6985940

[165] Fish F Ziff M. The *In Vitro* and *In Vivo* Induction of Anti-Double-Stranded DNA Antibodies in Normal and Autoimmune Mice. J Immunol (1982) 128(1):409–14. 6976378

[166] Swanson PC Ackroyd C Glick GD. Ligand Recognition by Anti-DNA Autoantibodies. Affinity, Specificity, and Mode of Binding. Biochemistry (1996) 35(5):1624–33. doi: 10.1021/bi9516788 8634294

[167] Desai NA Shankar V. Single-Strand-Specific Nucleases. FEMS Microbiol Rev (2003) 26(5):457–91. doi: 10.1111/j.1574-6976.2003.tb00626.x 12586391

[168] Herbert A Rich A. The Biology of Left-Handed Z-DNA. J Biol Chem (1996) 271(20):11595–8. doi: 10.1074/jbc.271.20.11595 8662853

[169] Ravichandran S Subramani VK Kim KK. Z-DNA in the Genome: From Structure to Disease. Biophys Rev (2019) 11(3):383–7. doi: 10.1007/s12551-019-00534-1 PMC6557933 31119604

[170] Herbert A. Z-DNA and Z-RNA in Human Disease. Commun Biol (2019) 2:7. doi: 10.1038/s42003-018-0237-x 30729177 PMC6323056

[171] Wittig B Dorbic T Rich A. Transcription Is Associated With Z-DNA Formation in Metabolically Active Permeabilized Mammalian Cell Nuclei. Proc Natl Acad Sci USA (1991) 88(6):2259–63. doi: 10.1073/pnas.88.6.2259 PMC51210 2006166

[172] Ha SC Lowenhaupt K Rich A Kim YG Kim KK. Crystal Structure of a Junction Between B-DNA and Z-DNA Reveals Two Extruded Bases. Nature (2005) 437(7062):1183–6. doi: 10.1038/nature04088 16237447

[173] Rich A Zhang S. Timeline: Z-DNA: The Long Road to Biological Function. Nat Rev Genet (2003) 4(7):566–72. doi: 10.1038/nrg1115 12838348

[174] Wong B Chen S Kwon JA Rich A. Characterization of Z-DNA as a Nucleosome-Boundary Element in Yeast Saccharomyces Cerevisiae. Proc Natl Acad Sci USA (2007) 104(7):2229–34. doi: 10.1073/pnas.0611447104 PMC1892989 17284586

[175] Mulholland N Xu Y Sugiyama H Zhao K. SWI/SNF-Mediated Chromatin Remodeling Induces Z-DNA Formation on a Nucleosome. Cell Biosci (2012) 2:3. doi: 10.1186/2045-3701-2-3 22264354 PMC3293710

[176] Lafer EM Valle RP Moller A Nordheim A Schur PH Rich A. Z-DNA-Specific Antibodies in Human Systemic Lupus Erythematosus. J Clin Invest (1983) 71(2):314–21. doi: 10.1172/jci110771 PMC436869 6822666

[177] Lafer EM Moller A Nordheim A Stollar BD Rich A. Antibodies Specific for Left-Handed Z-DNA. Proc Natl Acad Sci USA (1981) 78(6):3546–50. doi: 10.1073/pnas.78.6.3546 PMC319606 6943554

[178] Stollar BD. The Experimental Induction of Antibodies to Nucleic Acids. Methods Enzymol (1980) 70(A):70–85. doi: 10.1016/s0076-6879(80)70042-3 6158656

[179] Brigido MM Stollar BD. Two Induced Anti-Z-DNA Monoclonal Antibodies Use VH Gene Segments Related to Those of Anti-DNA Autoantibodies. J Immunol (1991) 146(6):2005–9. 1900879

[180] Rekvig OP Moens U Sundsfjord A Bredholt G Osei A Haaheim H. Experimental Expression in Mice and Spontaneous Expression in Human SLE of Polyomavirus T-Antigen. A Molecular Basis for Induction of Antibodies to DNA and Eukaryotic Transcription Factors. J Clin Invest (1997) 99(8):2045–54. doi: 10.1172/JCI119373 PMC508030 9109450

[181] Shlyakhtenko LS Potaman VN Sinden RR Lyubchenko YL. Structure and Dynamics of Supercoil-Stabilized DNA Cruciforms. J Mol Biol (1998) 280(1):61–72. doi: 10.1006/jmbi.1998.1855 9653031

[182] Kurahashi H Inagaki H Yamada K Ohye T Taniguchi M Emanuel BS. Cruciform DNA Structure Underlies the Etiology for Palindrome-Mediated Human Chromosomal Translocations. J Biol Chem (2004) 279(34):35377–83. doi: 10.1074/jbc.M400354200 PMC2810964 15208332

[183] Inagaki H Ohye T Kogo H Tsutsumi M Kato T Tong M. Two Sequential Cleavage Reactions on Cruciform DNA Structures Cause Palindrome-Mediated Chromosomal Translocations. Nat Commun (2013) 4:1592. doi: 10.1038/ncomms2595 23481400

[184] Frappier L Price GB Martin RG Zannis-Hadjopoulos M. Monoclonal Antibodies to Cruciform DNA Structures. J Mol Biol (1987) 193(4):751–8. doi: 10.1016/0022-2836(87)90356-1 3612792

[185] Brown BA Li Y Brown JC Hardin CC Roberts JF Pelsue SC. Isolation and Characterization of a Monoclonal Anti-Quadruplex DNA Antibody From Autoimmune "Viable Motheaten" Mice. Biochemistry (1998) 37(46):16325–37. doi: 10.1021/bi981354u 9819225

[186] Doaty S Agrawal H Bauer E Furst DE. Infection and Lupus: Which Causes Which? Curr Rheumatol Rep (2016) 18(3):13. doi: 10.1007/s11926-016-0561-4 26951251

[187] Marion TN Postlethwaite AE. Chance, Genetics, and the Heterogeneity of Disease and Pathogenesis in Systemic Lupus Erythematosus. Semin Immunopathol (2014) 36(5):495–517. doi: 10.1007/s00281-014-0440-x 25102991

[188] Thiyagarajan D Fismen S Seredkina N Jacobsen S Elung-Jensen T Kamper AL. Silencing of Renal DNaseI in Murine Lupus Nephritis Imposes Exposure of Large Chromatin Fragments and Activation of Toll Like Receptors and the Clec4e. PloS One (2012) 7(3):e34080. doi: 10.1371/journal.pone.0034080 22479529 PMC3316608

[189] Munoz LE Herrmann M. When Autologous Chromatin Becomes a Foe. Autoimmunity (2012) 45(8):565–7. doi: 10.3109/08916934.2012.719949 22978407

[190] Gupta S Kaplan MJ. The Role of Neutrophils and NETosis in Autoimmune and Renal Diseases. Nat Rev Nephrol (2016) 12(7):402–13. doi: 10.1038/nrneph.2016.71 PMC5510606 27241241

[191] Berger A. Hypersensitivity Revisited. BMJ (1998) 317(7166):1110. doi: 10.1136/bmj.317.7166.1110 9784443 PMC1114106

[192] Hedberg A Mortensen ES Rekvig OP. Chromatin as a Target Antigen in Human and Murine Lupus Nephritis. Arthritis Res Ther (2011) 13(2):214. doi: 10.1186/ar3281 21542875 PMC3132027

[193] Rekvig OP Thiyagarajan D Pedersen HL Horvei KD Seredkina N. Future Perspectives on Pathogenesis of Lupus Nephritis: Facts, Problems, and Potential Causal Therapy Modalities. Am J Pathol (2016) 186(11):2772–82. doi: 10.1016/j.ajpath.2016.06.026 27664472

[194] Mjelle JE Kalaaji M Rekvig OP. Exposure of Chromatin and Not High Affinity for dsDNA Determines the Nephritogenic Impact of anti-dsDNA Antibodies in (NZBxNZW)F1 Mice. Autoimmunity (2009) 42(2):104–11. doi: 10.1080/08916930802375729 19005880

[195] Chan OT Hannum LG Haberman AM Madaio MP Shlomchik MJ. A Novel Mouse With B Cells But Lacking Serum Antibody Reveals an Antibody-Independent Role for B Cells in Murine Lupus. J Exp Med (1999) 189(10):1639–48. doi: 10.1084/jem.189.10.1639 PMC2193634 10330443

[196] Waldman M Madaio MP. Pathogenic Autoantibodies in Lupus Nephritis. Lupus (2005) 14(1):19–24. doi: 10.1191/0961203305lu2054oa 15732283

[197] LeBlanc BA Urowitz MB Gladman OD. Serologically Active, Clinically Quiescent Systemic Lupus Erythematosus–Longterm Followup. J Rheumatol (1994) 21(1):174–5. 8151579

[198] Gladman DD Urowitz MB Keystone EC. Serologically Active Clinically Quiescent Systemic Lupus Erythematosus: A Discordance Between Clinical and Serologic Features. Am J Med (1979) 66(2):210–5. doi: 10.1016/0002-9343(79)90529-1 218447

[199] Fenton K Fismen S Hedberg A Seredkina N Fenton C Mortensen ES. Anti-dsDNA Antibodies Promote Initiation, and Acquired Loss of Renal Dnase1 Promotes Progression of Lupus Nephritis in Autoimmune (NZBxNZW)F1 Mice. PloS One (2009) 4(12):e8474. doi: 10.1371/journal.pone.0008474 20041189 PMC2793523

[200] Seredkina N van d V Berden J Mortensen E Rekvig OP. Lupus Nephritis: Enigmas, Conflicting Models and an Emerging Concept. Mol Med (2013) 19:161–9. doi: 10.2119/molmed.2013.00010 PMC3745592 23752208

[201] Seredkina S Rekvig OP. Acquired Loss of Renal Nuclease Activity Is Restricted to DNaseI and Is an Organ-Selective Feature in Murine Lupus Nephritis. Am J Pathol (2011) 79:1120–8. doi: 10.1016/j.ajpath.2011.05.011 PMC3157182 21723244

[202] Zykova SN Tveita AA Rekvig OP. Renal Dnase1 Enzyme Activity and Protein Expression Is Selectively Shut Down in Murine and Human Membranoproliferative Lupus Nephritis. PloS One (2010) 5(8). doi: 10.1371/journal.pone.0012096 PMC2938370 20856893

[203] Wang X Xia Y. Anti-Double Stranded DNA Antibodies: Origin, Pathogenicity, and Targeted Therapies. Front Immunol (2019) 10:1667. doi: 10.3389/fimmu.2019.01667 31379858 PMC6650533

[204] Goldberg BS Ackerman ME. Antibody-Mediated Complement Activation in Pathology and Protection. Immunol Cell Biol (2020) 98(4):305–17. doi: 10.1111/imcb.12324 PMC7293394 32142167

[205] Sharp TH Boyle AL Diebolder CA Kros A Koster AJ Gros P. Insights Into IgM-Mediated Complement Activation Based on *in Situ* Structures of IgM-C1-C4b. Proc Natl Acad Sci USA (2019) 116(24):11900–5. doi: 10.1073/pnas.1901841116 PMC6575175 31147461

[206] Bajic G Degn SE Thiel S Andersen GR. Complement Activation, Regulation, and Molecular Basis for Complement-Related Diseases. EMBO J (2015) 34(22):2735–57. doi: 10.15252/embj.201591881 PMC4682646 26489954

[207] Villalta D Bizzaro N Bassi N Zen M Gatto M Ghirardello A. Anti-dsDNA Antibody Isotypes in Systemic Lupus Erythematosus: IgA in Addition to IgG anti-dsDNA Help to Identify Glomerulonephritis and Active Disease. PloS One (2013) 8(8):e71458. doi: 10.1371/journal.pone.0071458 23951169 PMC3741383

[208] Gronwall C Akhter E Oh C Burlingame RW Petri M Silverman GJ. IgM Autoantibodies to Distinct Apoptosis-Associated Antigens Correlate With Protection From Cardiovascular Events and Renal Disease in Patients With SLE. Clin Immunol (2012) 142(3):390–8. doi: 10.1016/j.clim.2012.01.002 PMC3632049 22297166

[209] Tsokos GC. Systemic Lupus Erythematosus. N Engl J Med (2011) 365(22):2110–21. doi: 10.1056/NEJMra1100359 22129255

[210] Lackman D Mudd S Sevag MG Smolens J Wiener M. The Serological Reactivity of Nucleic Acid. J Immunol (1941) 40:1–20.

[211] Gay D Saunders T Camper S Weigert M. Receptor Editing: An Approach by Autoreactive B Cells to Escape Tolerance. J Exp Med (1993) 177(4):999–1008. doi: 10.1084/jem.177.4.99 8459227 PMC2190958

[212] Radic MZ Erikson J Litwin S Weigert M. B Lymphocytes may Escape Tolerance by Revising Their Antigen Receptors. J Exp Med (1993) 177(4):1165–73. doi: 10.1084/jem.177.4.1165 PMC2190988 8459210

[213] Weigert MG Cesari IM Yonkovich SJ Cohn M. Variability in the Lambda Light Chain Sequences of Mouse Antibody. Nature (1970) 228(5276):1045–7. doi: 10.1038/2281045a0 5483159

[214] Jang YJ Stollar BD. Anti-DNA Antibodies: Aspects of Structure and Pathogenicity. Cell Mol Life Sci (2003) 60(2):309–20. doi: 10.1007/s000180300026 PMC11138676 12678496

[215] Schwartz RS. Genetic and Environmental Issues in Assessing the Role of DNA Antibodies–a Summary of Research Presented at the DNA Antibody Workshop. Lupus (1997) 6(3):344–5. doi: 10.1177/096120339700600332 9296786

[216] Rahman A Isenberg DA. Systemic Lupus Erythematosus. N Engl J Med (2008) 358(9):929–39. doi: 10.1056/NEJMra071297 18305268

[217] Isenberg DA Manson JJ Ehrenstein MR Rahman A. Fifty Years of Anti-Ds DNA Antibodies: Are We Approaching Journey’s End? Rheumatol (Oxford) (2007) 46(7):1052–6. doi: 10.1093/rheumatology/kem112 17500073

[218] Tsokos GC Lo MS Costa RP Sullivan KE. New Insights Into the Immunopathogenesis of Systemic Lupus Erythematosus. Nat Rev Rheumatol (2016) 12(12):716–30. doi: 10.1038/nrrheum.2016.186 27872476

[219] Pisetsky DS. The Complex Role of DNA, Histones and HMGB1 in the Pathogenesis of SLE. Autoimmunity (2014) 47(8):487–93. doi: 10.3109/08916934.2014.921811 PMC4410020 24916843

[220] Schroeder K Herrmann M Winkler TH. The Role of Somatic Hypermutation in the Generation of Pathogenic Antibodies in SLE. Autoimmunity (2013) 46:121–7. doi: 10.3109/08916934.2012.748751 23181829

[221] Muller S Radic M. Oxidation and Mitochondrial Origin of NET DNA in the Pathogenesis of Lupus. Nat Med (2016) 22(2):126–7. doi: 10.1038/nm.4044 26845404

[222] Lacotte S Dumortier H Decossas M Briand JP Muller S. Identification of New Pathogenic Players in Lupus: Autoantibody-Secreting Cells Are Present in Nephritic Kidneys of (NZBxNZW)F1 Mice. J Immunol (2010) 184(7):3937–45. doi: 10.4049/jimmunol.0902595 20181885

[223] Dieker JW Fransen JH van Bavel CC Briand JP Jacobs CW Muller S. Apoptosis-Induced Acetylation of Histones Is Pathogenic in Systemic Lupus Erythematosus. Arthritis Rheum (2007) 56(6):1921–33. doi: 10.1002/art.22646 17530637

[224] Monneaux F Muller S. Epitope Spreading in Systemic Lupus Erythematosus: Identification of Triggering Peptide Sequences. Arthritis Rheum (2002) 46(6):1430–8. doi: 10.1002/art.10263 12115171

